# Nicotinamide-Loaded Peptoid Nanotubes for Energy Regeneration in Acute Brain Injury

**DOI:** 10.1021/acsnano.5c19897

**Published:** 2026-04-13

**Authors:** Hui Du, Thi Kim Hoang Trinh, Olivia C. Brandon, Renyu Zheng, Haoyu Wang, Kylie Corry, Thomas R. Wood, Chun-Long Chen, Elizabeth Nance

**Affiliations:** † Department of Chemical Engineering, 7284 University of Washington, Seattle Washington 98195, United States; ‡ Physical Sciences Division, 6865 Pacific Northwest National Laboratory, Richland, Washington 99352, United States; § Division of Neonatology, Department of Pediatrics, 7284 University of Washington, Seattle, Washington 98195, United States; ∥ Department of Bioengineering, University of Washington, Seattle, Washington 98195, United States; ⊥ Department of Radiology, University of Washington, Seattle, Washington 98195, United States

**Keywords:** ATP, cell metabolism, neonatal, drug delivery, microglia, poly-N-substituted glycines

## Abstract

Acute brain injuries such as perinatal asphyxia, stroke, and traumatic brain injury result in ischemia, oxidative stress, excitotoxicity, and inflammation, leading to a depletion of ATP. Nicotinamide adenine dinucleotide (NAD+) is crucial for ATP regeneration and DNA repair during postinjury recovery. However, the therapeutic benefits of NAD+ and its precursors, such as nicotinamide (NAM), are limited by challenges in achieving effective cell-specific intracellular delivery. In this study, we use a nanopeptoid delivery strategy to replenish the cellular redox state and increase energy production in the acutely injured brain. By self-assembling peptoids into tubular structures, we created biocompatible NAM-conjugated peptoid nanotubes (NAM-PNTs) that vary in tubular length. NAM-PNTs demonstrated significant therapeutic benefits by enhancing cell viability and replenishing intracellular ATP levels within 24 h of treatment in oxygen–glucose-deprived (OGD) BV-2 cells. In organotypic brain slices, NAM-PNT treatment promoted glial proliferation, reduced proinflammatory cytokines, and increased anti-inflammatory cytokines after OGD, an *ex vivo* model of hypoxia-ischemia. The effect of NAM-PNTs is associated with their uptake into microglia via fluid-phase phagocytosis and caveolae-mediated endocytosis. A single systemic dose of NAM-PNTs localized in microglia in the injured hemisphere and reduced brain tissue loss and improved neuropathology after hypoxia-ischemia in term-equivalent rats. These findings highlight the therapeutic potential of NAM-PNTs for cell-specific targeted delivery and energy restoration in the acutely injured neonatal brain. In the neonatal brain injury field, this work demonstrates the development of an innovative nanoparticle platform from first-principles design and synthesis to *in vitro* screening and then demonstration of efficacy *in vivo*.

## Graphical abstract

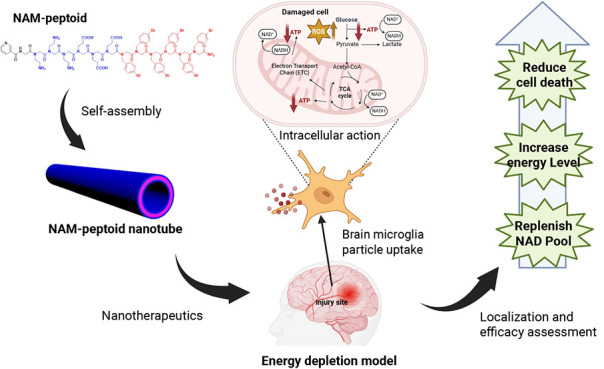

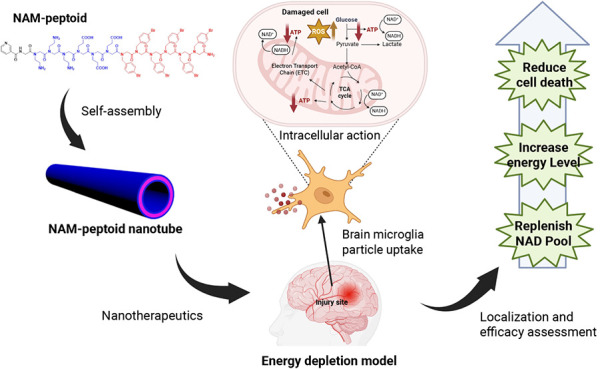

The brain is the body’s most metabolically active tissue and depends on the continuous delivery of nutrients and energy sources, including glucose and oxygen, supplied by cerebral blood flow. Cellular respiration occurs via glycolysis, the tricarboxylic acid (TCA) cycle, and oxidative phosphorylation, where glucose is consumed to generate adenosine triphosphate (ATP), the primary cellular energy source.^1,2^ Nicotinamide adenine dinucleotide (NAD+) acts as an essential coenzyme in ATP production through its ability to accept hydride equivalents, forming NADH that provides electrons to the electron transport chain (ETC) to generate most ATP.^3^

Both ATP and NAD+ levels can be affected by factors including age and health status.^4,5^ ATP and NAD+ levels in the brain are also impacted by acute injuries such as stroke, traumatic brain injury (TBI), and perinatal asphyxia, which lead to ischemic, excitotoxic, and inflammatory processes.^6^ These pathological processes result in mitochondrial dysfunction that reduces glycolysis and increases oxidative stress that drives DNA damage.^4^ DNA damage triggers cell death through activation of the poly­(ADP-ribose) polymerase-1 (PARP-1) cell death pathway, which consumes NAD+ to repair damaged DNA. Rapid consumption of NAD+ further diminishes cellular ATP production, causing energy depletion and further cell death.^7,8^ One potential way to mitigate energy depletion after injury is to directly deliver NAD+ to injured cells. For example, in a rat model of transient focal brain ischemia, intranasal administration of NAD+ decreased brain damage.^9^ Supplementation of NAD+ has also been shown to ameliorate neuropathological defects and markedly extend lifespan by promoting DNA repair and improving mitochondrial function in models of Ataxia Telangiectasia and Alzheimer’s disease (AD), decreasing neurodegenerative processes.^10–13^ Nicotinamide adenine dinucleotide (NMN), a precursor of NAD+, has been shown to have neuroprotective effects in hypoxia–ischemia^14^ and can also prevent the increase in PAR formation and NAD+ catabolism, reducing brain injury.^15^

Despite the neuroprotective potential of restoring brain NAD+ levels after injury, several issues remain to be addressed. In many studies, high and frequent doses of NAD+ and NMN are required to see a benefit. In addition, NAD+ cannot pass through the cellular membrane,^16^ and the cell-specific targeting of NAD+ is limited. Exogenous NAD+ is likely to be metabolized by extracellular nucleotidases in the bloodstream, and the conversion rate in the brain of the precursors nicotinamide (NAM), NMN, and nicotinamide riboside (NR) to NAD+ remains poorly understood.^17^ Recent evidence suggests that NAD+ in the brain is more likely to be made by NAM-derived NMN available locally to brain cells, rather than from NR or NMN given via intravenous injection or oral delivery.^17^ Delivery vehicles could be used to reduce dose and dosing frequency, as well as increase cell specificity and protection from enzymatic degradation, in order to facilitate the import of NAD+ or NAD+ precursors into damaged brain cells.

Nanomaterials are promising delivery vehicles for the intracellular drug delivery of NAD+ or NAD+ precursors. In hepatocytes, quantum dots (QDs) conjugated with NMN achieved a comparable treatment effect with a lower dose compared to free NMN.^18^ Lipid metal–organic framework (MOF) nanomaterials enabled NAD­(H) to be delivered into the cellular compartment directly, replenishing the cellular NAD+ pool and increasing the survival rate after sepsis.^19^ However, toxicological studies using nanomaterials like QDs and MOFs have shown potential acute and chronic hazards to humans.^2,20^ In addition, there are no current studies on the use of biocompatible nanomaterials for the delivery of NAD+ or its precursors to specific cells in the brain.

In this study, we utilize nanotubes assembled from sequence-defined peptoids, or poly-N-substituted glycines, covalently attached with NAM to replenish NAD+ levels in the acutely injured, energy-depleted brain. Peptoids are well-advanced sequence-defined synthetic polymers that have advantages of synthetic polymers and biopolymers and can be used as protein mimetics.^21–23^ A wide selection of amines can result in large side-chain diversity while exhibiting protein-like molecular recognition for various applications.^21,23–25^ Peptoids do not have backbone hydrogen bond donors, and therefore offer the unique simplicity for controlled assembly into nanomaterials by precisely tuning peptoid–peptoid and peptoid–surface interactions through the variation of side chains.^23,25,26^ A large number of amphiphilic peptoids have been designed and synthesized for their assembly into hierarchically structured crystalline nanomaterials with a broad range of architectures,^21,23,25^ including nanotubes.^27^

Peptoid nanotubes (PNTs) are promising biosensing, bioimaging, and drug delivery platforms.^27–30^ Due to their protein-like sequence-specific molecular recognition, PNTs experience high stability and biocompatibility in cells and can accumulate in hard-to-target organs,^28,30,31^ criteria that can be promising for brain delivery. Because nanotubes with varied lengths can extravasate more readily and easily penetrate through the tissue parenchyma and cell membrane,^32–35^ herein, we synthesized NAM-conjugated peptoid nanotubes (NAM-PNTs) and NAD+-associated PNTs of different lengths with high drug-loading efficiency. We demonstrated no cytotoxicity in brain cells or organotypic whole hemisphere (OWH) brain slices. We showed that NAM-PNTs improved cellular protection compared to free drugs by replenishing cellular ATP levels and the NAD+ pool after acute injury in oxygen–glucose-deprivation (OGD)-mediated injury. After acute *in vivo* hypoxia-ischemia (HI), NAM-PNTs significantly reduced brain area loss and downregulated inflammatory cytokine responses. Our study demonstrates that NAM delivery via PNTs is therapeutically promising for cell-specific delivery that results in energy regeneration in the acutely injured brain.

## Results and Discussion

### Formulation and Characterization of Tunable and Stable NAM-PNTs

Employing a previously established solid-phase submonomer synthesis method,^36,37^ we designed and synthesized tube-forming peptoids, both with and without drug and dye molecules. Illustrated in Figure 1a, these peptoid sequences consist of three N-(2-aminoethyl) glycine (Nae) and N-(2-carboxyethyl) glycine (Nce) groups forming the polar domain alongside six N-[(4-bromophenyl)­methyl] glycine (Nbrpm) groups comprising the hydrophobic domain. Conjugation of drugs, including nicotinic acid to conjugate NAM or thymine-1-acetic acid (Thy) to electrostatically associate NAD+, and dye molecules such as dansyl (DNS), occurred at the N-terminus adjacent to the polar domain. A detailed account of the preparation, purification, and characterization of these four peptoids (Pep-H: Nbrpm6Nce3Nae3; Pep-NAM: Nbrpm6Nce3Nae3Nc2NAM, Pep-Thy: Nbrpm6Nce3Nae3Nc2Thy, and Pep-DNS: Nbrpm6Nce3Nae3Nc2DNS) is provided in the Methods section and Supporting Information (Figures S1–S4). PNTs were assembled using a method similar to evaporation-induced crystallization.^30,38^ Figure S5 displays both atomic force microscopy (AFM) and scanning electron microscopy (SEM) data, indicating that nanotubes assembled from Pep-H, Pep-NAM, Pep-Thy, and Pep-DNS exhibit a similar structure. Ex-situ AFM results indicated heights of 6.53 ± 0.29 (Pep-H), 7.04 ± 0.32 (Pep-NAM), 14.93 ± 1.25 (Pep-Thy), and 7.78 ± 0.07 nm (Pep-DNS). X-ray diffraction (XRD) data verified that these PNTs are highly crystalline, displaying similar XRD patterns to those PNTs we previously reported (Figure 1b).^27–30^ The peak at 1.67 nm spacing signifies the separation between two peptoid backbones aligned with the hydrophobic Nbrpm groups facing each other.^27,28,39,40^ This observed spacing of 5.71 Å is attributed to the meticulous arrangement of aromatic side chains, specifically Nbrpm6. Additionally, a 4.61 Å spacing corresponds to the alignment of lipid-like peptoid chains. Further, the peaks at 4.32 Å, 3.81 Å, and 3.35 Å suggest extensive π–π stacking interactions.^41–43^ Consistent with our prior work,^27^ the hydrophobic Nbrpm groups are densely packed together and intricately positioned at the core of the nanotube wall. The polar Nae and Nce groups are located on both surfaces of the nanotube, exhibiting a packing similar to PNTs reported previously.^27^ These results demonstrate that drug and dye molecules can be precisely incorporated within crystalline nanotubes with a tunable density while maintaining a consistent tubular framework structure.

**Figure 1:**
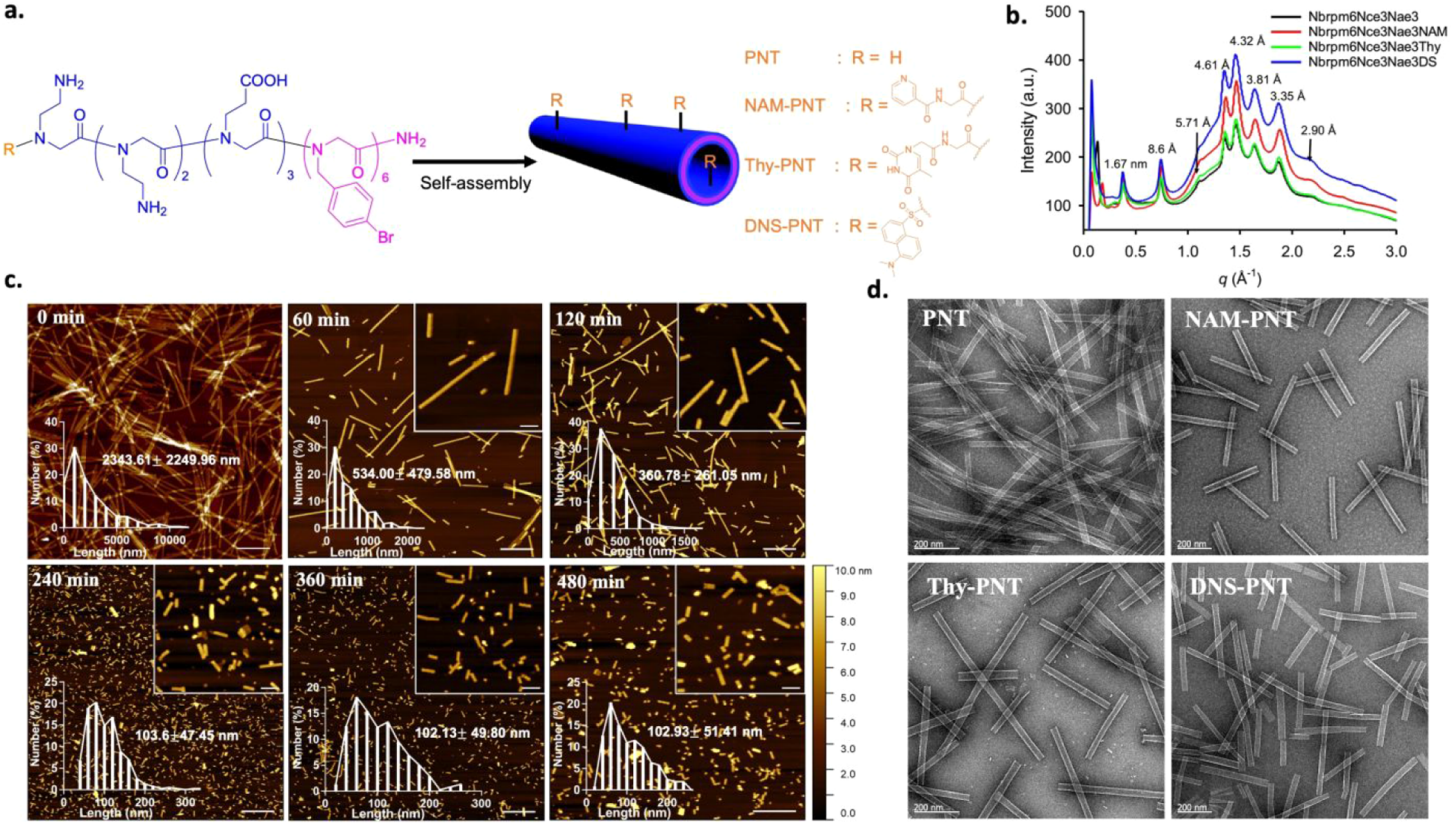
Characterizations of the PNT, NAM-PNT, Thy-PNT, and DNS-PNT. a, Schematic illustration of the peptoid structure and self-assembly. R indicates four different end groups in the peptoid. b, XRD data of PNT, NAM-PNT, Thy-PNT, and DNS-PNT showing similar crystalline structures for all peptoids. c, AFM images showing NAM-PNTs with various lengths after 0, 60, 120, 240, 360, and 480 min of sonication. Scale bars: 1 μm and 200 nm (insets). d, TEM images of PNT, NAM-PNT, Thy-PNT, and DNS-PNT, respectively. Scale bar: 200 nm.

We further showed that NAM-PNTs remained structurally unchanged in PBS over 5 days (Figure S6). The structural stability of PNTs in a 0.2 wt % Pronase solution^44^ across different temperatures was evaluated to show the PNT enzymatic stability (Figure S7). PNTs remained intact for 10 days at 37 °C, showing some degree of increased aggregation over time. The lengths of PNTs can be adjusted through sonication. The length distribution of the PNTs at each sonication time was quantified (Figure S8) and the average lengths are reported in Table S1, showing a reduction in length with increasing water bath sonication time (Figure 1c). In addition to water bath sonication, probe sonication of PNTs can provide an alternative method to shorten the tube length and achieve greater monodispersity; representative TEM images of PNTs following probe sonication are shown in Figure 1d. Sonication can cut the PNTs by inducing cavitation bubbles that release intense localized energy in the form of shockwaves and high shear forces. For NAM-PNTs, sonication did not impact NAM-conjugate stability as no NAM was released even after 360 min of sonication (Figure S9).

### NAM-PNTs Show No Cytotoxicity in Healthy Brain Cells and Promote Energy Replenishment following Oxygen–Glucose Deprivation

To confirm that PNTs are biocompatible, NAM-PNTs and Thy-PNTs were tested in BV-2 cells. Dose-dependent effects on cell viability and cytotoxicity were explored with exposure to 2, 20, and 50 μg/mL of PNTs. Both formulations retained high cell viability for all three dosages over 24 h of treatment (Figure 2a,b), indicating that PNTs have no toxic effect on healthy cells, regardless of cell seeding density (Figure S10). This result was anticipated since peptoids are known to have protein-like sequence-specific molecular recognition due to their similar structure to peptides, but confirmation in brain cells is important for the intended application. For NAM-PNTs specifically, all dosages increased cell viability. At 50 μg/mL, NAM-PNTs provided a nonsignificant increase (11.3%) in cell viability in healthy cells compared to cells not exposed to PNTs (Figure 2a). However, this increase was not seen in the healthy cells treated with free NAM or NAD+ (Figure S11). In comparison, Thy-PNTs showed a nonsignificant decrease in cell viability with increasing Thy-PNT dose (Figure 2b).

**Figure 2:**
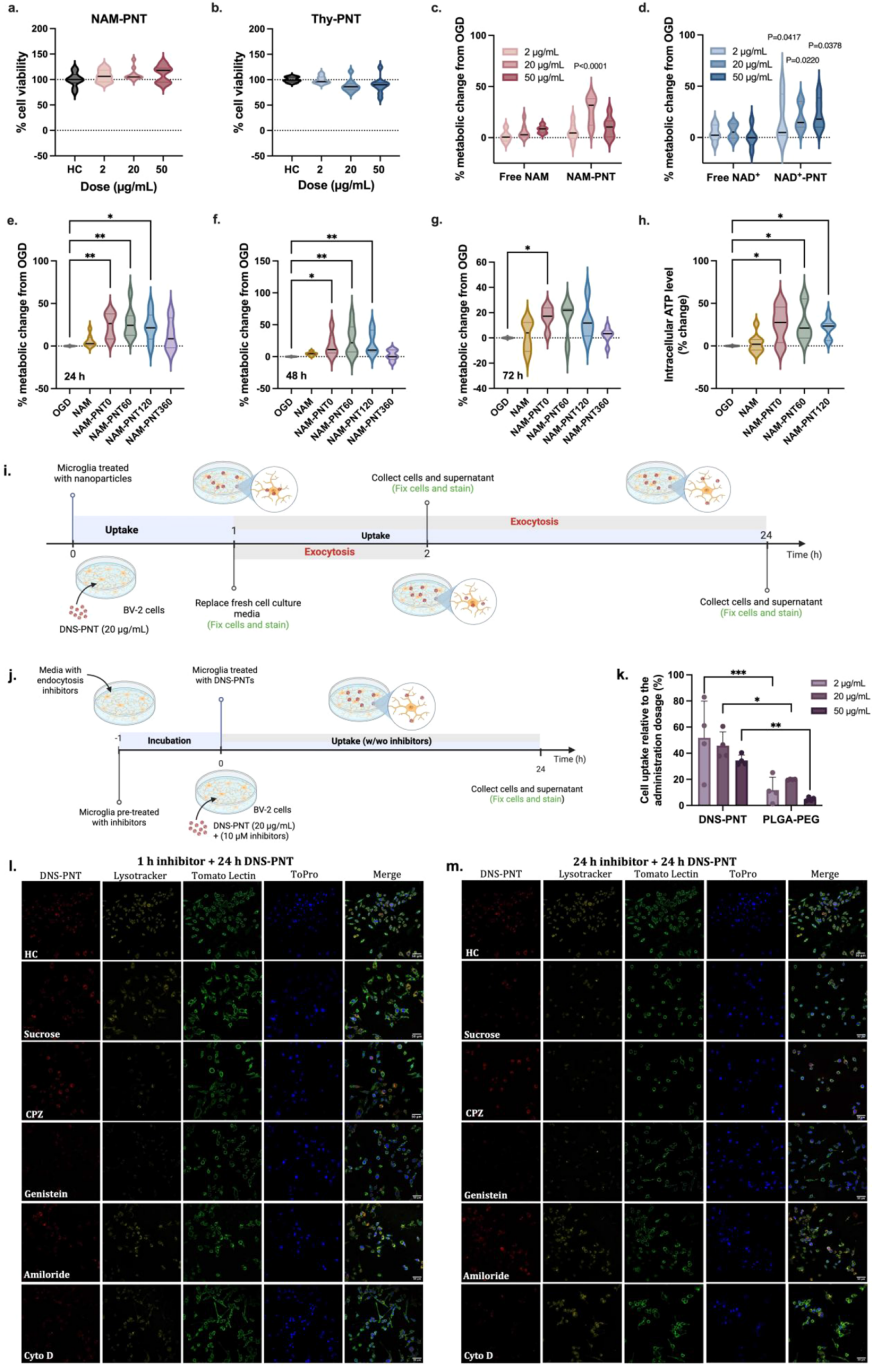
NAM-PNTs show high biocompatibility and improve the cellular energy and metabolic state in the energy-depleted, acutely injured brain. a,b, MTT data showing that both NAM-PNTs (a) and Thy-PNTs (b) result in negligible effects on cell viability compared to healthy controls at all three dosages. (*n* = 10). c,d, BV-2 cells exposed to 10 min of OGD showed improvement in cell viability (AlamarBlue assay) after treatment with NAM-PNTs (c) and NAD+-PNTs (d) compared to NAM and NAD+ in free form. Readings were normalized to OGD controls. Statistical significance was calculated via ordinary two-way ANOVA (*n* = 3–7). e,f,g, Sonication time impacts cell viability compared to nontreated OGD-exposed controls after exposure to 20 μg/mL of NAM-PNTs for 24 h (e), 48 h (f), and 72 h (g). Readings were normalized to OGD controls. Statistical significance was calculated via a Kruskal–Wallis test (*n* = 3–6). h, Intracellular ATP levels of OGD-exposed BV-2 cells with the addition of free NAM and NAM-PNTs compared to the nontreated control. All data are presented as violin plots that show the median with the interquartile range. Statistical significance was calculated via a Kruskal–Wallis test. i,j, Schematic illustration of nanoparticle uptake (h) and exocytosis (l) assays in BV-2 cells. Figure created with Biorender.com. k. BV-2 cell uptake of DNS-PNT and PLGA–PEG nanoparticles at different administration dosages after 1 h of endocytosis. Data are presented as mean ± s.d. and statistical significance was calculated via ordinary two-way ANOVA (*n* = 4). l,m, Confocal images showing DNS-PNTs (red) associated with lysosomes (Lysotracker, yellow) and BV-2 cells (tomato lectin, green; ToPro-3, blue) preincubated with inhibitors for 1 h and with (l) or without (m) inhibitors for 24 h.

We next sought to quantify the effect of NAM- or NAD+-PNTs after an acute injury that drives reduced glycolysis and oxidative respiration due to mitochondrial dysfunction.^45^ In this study, we applied OGD, an *in vitro* model of acute hypoxic-ischemic (HI) brain injury, to BV-2 cells. We first confirmed that OGD-exposed BV-2 cells showed decreased cell viability compared to healthy cells (Figure S12). In the cells exposed to OGD, the addition of NAM-PNTs increased metabolic activity, while free NAM did not (Figure 2c). A dose-dependent effect was also observed: 20 μg/mL NAM-PNTs led to the highest increase in metabolic activity, with minimal effect at 2 μg/mL and a possible reduction in benefit at 50 μg/mL.

Delivering NAD+ directly to cells could be another method to regulate disordered metabolic states as it can decrease PARP-1-mediated cell death by maintaining glycolysis and prevent mitochondrial depolarization and release of mitochondrial apoptosis-inducing factor.^7,46,47^ Several reports suggest that NAD+ can decrease neuronal death resulting from OGD-induced injury by enhancing DNA repair activity.^48,49^ However, the delivery of free NAD+ is limited, as NAD+ cannot pass through cellular membranes and has no known uptake transporters.^16^ To overcome this, NAD+ can be physically associated with Thy-PNTs via electrostatic interaction to achieve intracellular delivery. Treatment with NAD+-PNTs resulted in improvements in metabolic activity similar to those seen with NAM-PNTs (Figure 2d), though there was no dose-dependence of effect within the doses tested. By comparison, free NAD+ had no effect.

To further investigate the efficacy of NAM-PNTs and NAD+-PNTs, we analyzed the effect of PNT length, an important physical property of a nanotubular delivery system that can influence cellular uptake.^50^ PNTs were cut by applying sonication. Three sonication times of 0 (PNT0), 60 (PNT60), and 120 min (PNT120) were applied to both NAM-PNTs and NAD+-PNTs. For all three sonication times, 20 μg/mL was determined to be the optimal dose (Figure S13a). After 24 h of treatment, all three lengths of NAM-PNT showed comparable amounts of increase in metabolic activity compared with untreated OGD (Figure 2e). A similar trend was measured at 48 h after NAM-PNT treatment, which was sustained until 72 h (Figure 2f,g). However, we observed that NAM-PNT360 did not improve cell viability, suggesting decreased efficacy with short PNT lengths. In healthy cells, NAM-PNT480 even showed an adverse effect on cell viability (Figure S14), suggesting that much shorter PNTs may induce cell cytotoxicity, reducing overall cell viability. Unlike NAM-PNTs, the dose effect of NAD+-PNT was not clearly determined (Figure S13b) and the NAD+-PNTs showed no significant effect on metabolic change with PNT length (Figure S15). In comparison to the chemical conjugation of NAM to PNTs, NAD+ was electrostatically associated with the PNT through molecular interactions between adenine and thymine. This association may be disrupted upon cell association or internalization, which would result in the limited therapeutic efficacy observed with NAD+-PNT delivery.

Both free NAM and NAM-PNTs were able to enhance cellular ATP levels even in healthy cells over 24 h (Figure S16a). However, free NAM showed a limited increase compared to NAM-PNTs, with a 20 μg/mL dose of NAM-PNTs giving the highest increase in the cellular ATP level. By applying OGD to BV-2 cells, intracellular ATP levels decreased to 25.8% of the healthy state (Figure S16b). Three lengths of NAM-PNTs improved ATP levels by 27.6%, 20.9%, and 23.3%, respectively, after the OGD energy depletion, while the free NAM did not (Figure 2h). In an in vitro microglial cell line, we conclude that NAM-PNTs provide a larger therapeutic potential in energy-depleted cells compared to NAD+-PNTs, but the benefit is lost with the shortest PNT lengths.

With NAM-PNTs providing a high therapeutic effect in BV-2 cells, we further analyzed the PNT cellular uptake association at different administration dosages and compared it with PLGA–PEG nanoparticles, a well-developed drug delivery platform. To confirm that PNTs were associated with BV-2 cells, dansyl-conjugated PNTs (DNS-PNTs) were added to BV-2 cells for 1 h and then allowed to excrete particles for 1 or 24 h for the assessment of exocytosis (Figure 2i). Dansyl is a fluorescent material that can be detected with a high signal-to-noise ratio even in low concentrations and is widely conjugated to peptides via N-terminal substitution.^51^ Dansyl is covalently attached to the primary amino groups during PNT synthesis, self-assembling into DNS-PNTs. Compared to PLGA–PEG nanoparticles, DNS-PNTs showed rapid uptake, significantly greater cell association (Figure 2k), and slow cellular excretion across all doses (Figure S17).

To further explore the cellular uptake mechanism of how DNS-PNT gets into the BV-2 cells, we treated the cells with specific endocytic pathway inhibitors for 1 and 24 h and allowed DNS-PNT to associate with BV-2 cells for 24 h with or without inhibitors (Figure 2j). Cell viability with all inhibitors at different concentrations was confirmed, with 10 μM selected as the targeted concentration (Figure S18). The addition of amiloride (micropinocytosis inhibitor) and chlorpromazine (CPZ, clathrin-mediated endocytosis inhibitor) had a minimal impact on PNT internalization into BV-2 cells (Figure 2l, m). In contrast, sucrose (fluid-phase endocytosis) treatment for 24 h resulted in reduced PNT uptake. Notably, Cytochalasin D (Cyto D), which inhibits phagocytosis, and genistein, which inhibits caveolae-mediated endocytosis, significantly suppressed PNT signals as early as 1 h, with further decreases observed at 24 h. These findings suggest that phagocytosis and caveolae-mediated endocytosis are primary pathways for PNT to internalize into BV-2 cells. However, the detailed cellular mechanisms remain unclear, necessitating further study.

### Reduced Cell Death via NAM-PNT Treatment in OGD-Exposed Cultured Brain Slices

To capture the biocompatibility, efficacy, and cellular interaction of PNTs in a more complex model, we next evaluated NAM-PNTs in organotypic whole hemisphere (OWH) brain slices. OWH slices maintain 3D cytoarchitecture, a critical feature when trying to probe delivery into tissue, and retain functional relationships between cells.^52^ OWH brain slices also provide direct access to multiple brain regions, including the deep brain.^53^ Using OWH slices obtained from term-equivalent to human postnatal day 10 (P10) male rats,^52^ we topically applied the optimal dosage of 20 μg/mL of PNTs, as identified in our BV-2 studies, to OWH slices cultured for 4 days *in vitro* (DIV). We confirmed that PNTs do not negatively impact cell viability in the healthy OWH slice (Figure 3a). In fact, PNTs appear to decrease cytotoxicity compared to untreated healthy slices. When testing three different PNT lengths (NAM-PNT0, 60, and 120), we found that cytotoxicity decreases with decreasing PNT length, with PNT120 maintaining the highest cell viability, though this was not significant (p-trend = 0.227).

**Figure 3:**
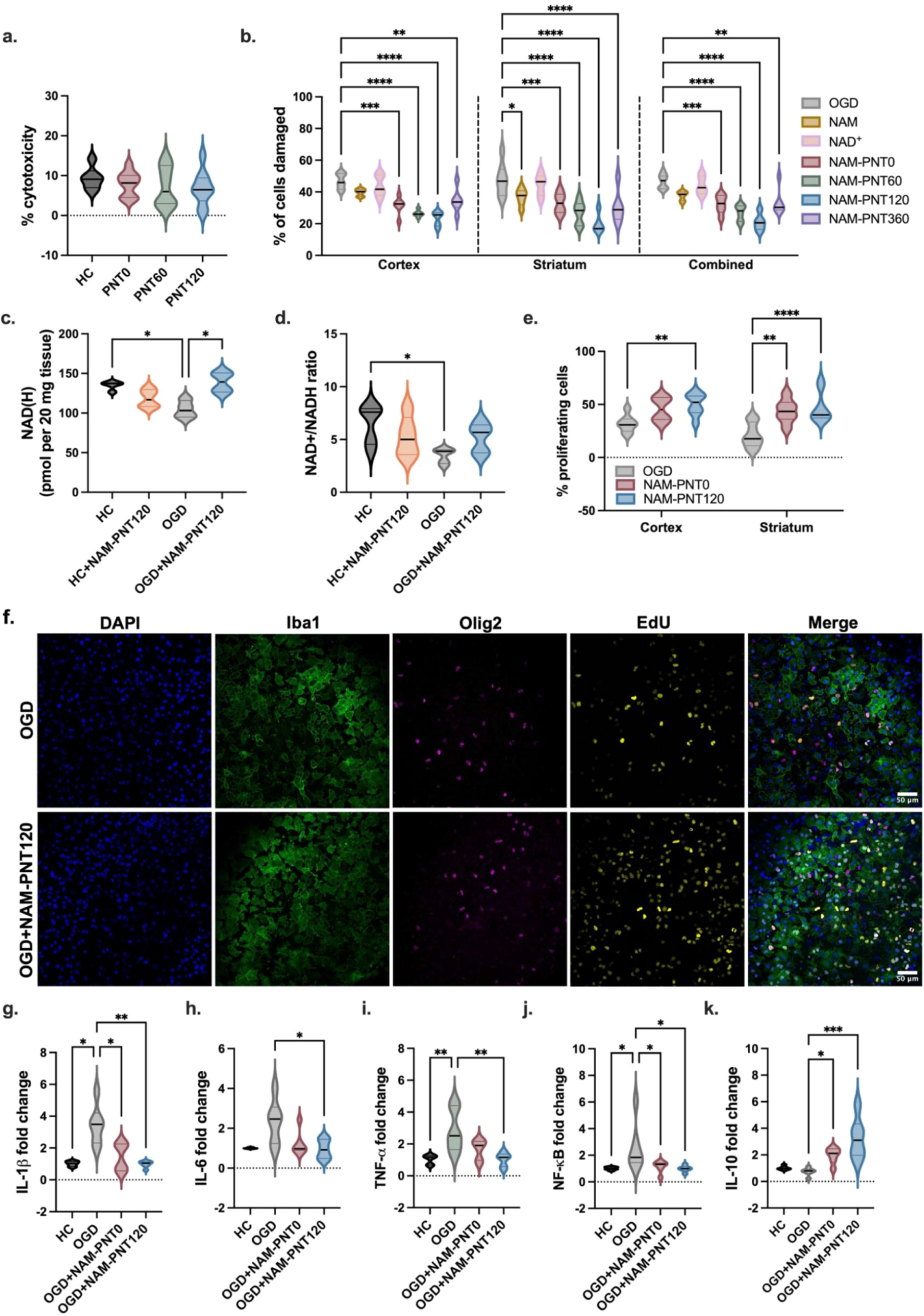
LDH, cell death and proliferation profile, and NAD levels of P10 brain slices. a, LDH showing low cytotoxicity of NAM-PNTs on healthy slices at different sonication times. LDH release (%) values are normalized to the LDH release of acute slices immediately treated with 10% TX-100 (*n* = 6–9). b, NAM-PNT treatment reduces the percentage of damaged cells, assessed by PI quantification, in a PNT length-dependent manner. P10 profiles are given for the cortex and the striatum, and all data are combined. Statistical significance was calculated via a Kruskal–Wallis test (*n* = 7). c,d, Intracellular NAD­(H) levels (c) and NAD+/NAD­(H) ratios (d) in healthy and OGD-exposed slices with or without NAM-PNT120 for 24 h. Statistical significance was calculated via a Kruskal–Wallis test (*n* = 5). e, Percentage of proliferating cells in P10 slices after 30 min of OGD at the cortex and the striatum. Statistical significance was calculated via ordinary two-way ANOVA (*n* = 6–8). f, Representative images of EdU (yellow), coated with Iba1 (green), Olig2 (magenta), and DAPI (blue). Scale bar: 50 μm. (g–k) Fold-changes of mRNA markers showing that NAM-PNTs help regulate RNA expression in OGD-exposed slices with PNT treatments. Primer sequences are defined in Table S2. Statistical significance was calculated via a Kruskal–Wallis test (*n* = 6). All data are presented as violin plots that show the full range with lines at the median with the interquartile range.

We next investigated the regional cellular response to NAM-PNT treatment in both the cortical and the striatal regions of the OGD-exposed OWH slices. The cortex and striatum are susceptible to hypoxic-ischemic events but are critical regions for normal brain function.^54^ In both brain regions, free NAM and free NAD+ did not improve cell viability compared to the OGD-exposed slices without treatment (Figure 3b), as evidenced by a high number of propidium iodide (PI)-positive cells (Figure S19). Nuclear PI accumulation is a common marker of cells undergoing necrosis.^55,56^ NAD+ is not able to permeate or transport through cellular membranes, and both free NAM and NAD+ are likely metabolized by extracellular nucleosidases rather than being directly taken up into brain cells.^17^ In the OGD-exposed slices, the percentage of PI+ cells was reduced for all NAM-PNT treatment conditions. For both the cortex and striatum, the relationship between cell death and PNT length showed that cell death decreases with shorter NAM-PNTs, with NAM-PNT120 treatment resulting in the lowest percentage of damaged cells, further supporting NAM-PNT120 as a promising platform for HI treatment.

The OWH slice model requires particles to traverse the extracellular space and the extracellular matrix. Prior literature has established that rod-like particles with a high aspect ratio, such as single-wall carbon nanotubes (SWCNTs) up to 700 nm in length, can readily diffuse in the brain cortex.^33^ Here, the therapeutic efficacy of NAM-PNTs is a trade-off with length: a higher aspect ratio contributes to cellular uptake while limiting the tissue penetration rate. NAM-PNT0 has an average length of 2 μm, which is too long to penetrate through tissue, leading to limited therapeutic efficacy. Though NAM-PNT60 showed the highest efficacy in BV-2 cells, PNT60 has a length of 500 nm with an aspect ratio of 14, which limits its distribution through the intact brain tissue of the OWH slice. With increasing sonication time, the length of PNTs is reduced, and the size distribution is more uniform. However, NAM-PNT360 did not show further improvement in efficacy compared to NAM-PNT120. PNTs that are too small, with a length of around 100 nm and an aspect ratio of 3, could be more rapidly cleared from the slice or have an altered extracellular and intracellular distribution that limits the effectiveness of NAM delivery.

To assess the direct effect of NAM-PNT on NAD+ and NADH levels in healthy and OGD-exposed slices, total NAD+ levels with and without NAM-PNT120 were quantified. While the addition of NAM-PNT120 did not affect healthy slices, the intracellular NAD­(H) levels of the OGD-exposed slices with NAM-PNT120 increased to levels similar to healthy slices (Figure 3c). NAM-PNT120 also improved the NAD+/NADH ratio in the OGD-exposed slices (p = 0.3441, Figure 3d) but had no effect on healthy slices. Under healthy conditions, NAD+ produced from exogenous NAM is more likely to be broken down into other metabolites to maintain the cellular redox state. However, when cells are NAD+ depleted, the introduction of NAM-PNT replenishes the NAD+ pool, increases the NAD+/NADH ratio, and reduces cell death.

### NAM-PNTs Drive Cell Proliferation, Reduce Proinflammatory Responses, and Restore DNA Damage Responses in the OGD-Exposed Brain

To further understand the beneficial effect of NAM-PNT120 on cellular responses to OGD, we measured cell proliferation via 5-ethynyl-2′-deoxyuridine (EdU) staining.^55,57,58^ In OGD-exposed slices, both NAM-PNT0 and NAM-PNT120 increased the number of EdU+ cells (Figure S20). This is consistent with the PI/DAPI data, as higher proliferation will result in lower relative cell death and higher metabolic activity. Regional differences in cellular proliferation were also assessed in the cortex and striatum (Figure 3e). In both regions, NAM-PNT120 led to a higher number of EdU+ cells. In NAM-PNT120-treated OGD slices, EdU+ signals were colocalized with cellular markers of both microglia and oligodendrocytes (Figure 3f).

OGD injury in the brain drives neuroinflammation and cell death through reduced oxidative respiration and increased oxidative stress.^59^ Before evaluating the impact of NAM-PNTs on inflammatory responses after OGD, we first applied NAM-PNT120 to healthy neonatal brain slices. We found that NAM-PNT120 resulted in no change in IL-10, TNF-α, and NF-κB, confirming that the PNTs do not drive an inflammatory response (Figure S21). In OGD-exposed slices, NAM-PNT120 was able to significantly reduce the production of IL-1β, IL-6, TNF-α, and NF-κB (Figure 3g,h,i, and j), while increasing IL-10 (Figure 3k), thus demonstrating a strong anti-inflammatory effect. The levels of each of these markers were returned to levels comparable to the healthy control. NAM-PNT0 also reduced proinflammatory cytokine production, but was less effective than NAM-PNT120, confirming previous results suggesting that NAM-PNT120 provides the most effective therapeutic efficacy.

Two prototypical cytokines that are widely explored after acute brain injury are IL-6 and IL-10. IL-6 has both pro- and anti-inflammatory effects with a central role in the integrated immune defense network against infections, but in the acute phase it is generally considered to be part of the proinflammatory injury response.^60^ IL-6 expression was increased in OGD-exposed slices and returned to the level seen in healthy slices by NAM-PNT0 and NAM-PNT120 (Figure 3h). The anti-inflammatory cytokine IL-10 was also significantly increased compared to the OGD control group after 24 h of exposure to NAM-PNTs (Figure 3k), suggesting that PNTs play a role in alleviating inflammation in injured tissues by upregulating IL-10 mRNA expression, driving an anti-inflammatory response.

Poly­(ADP-ribose) polymerase (PARP), which drives the consumption of NAD+, is associated with DNA damage.^61^ PARP-1 hydrolyzes NAD+, providing an ADP-ribose moiety that is polymerized into poly­(ADP-ribose) (PAR) in response to DNA-strand breaks.^62,63^ PAR regulates the maintenance of DNA integrity, gene expression, and cell division, and levels of PAR may act as a signature of normal DNA repair dynamics. An anti-PAR binding reagent was applied to qualitatively identify the effect of NAM-PNTs on PAR levels. Healthy slices had clear and strong PAR signals colocalizing with the nucleus, whereas the intensity of PAR was reduced in the OGD-exposed slices with a more diffuse, non-nuclear signal (Figure S22). When PARP-1 is activated in injured cells, NAD+ can be quickly consumed to aid in DNA repair, resulting in NAD pool depletion with the associated limitation of PAR production and cell death. NAM-PNT120 treatment after OGD increased the nuclear PAR signal, although some diffuse non-nuclear PAR signal remained (Figure S22). Further mechanistic studies are needed to determine the degree to which NAM-PNT120 initiates DNA repair mechanisms by replenishing the NAD+ pool.

### NAM-PNT Treatment Reduces Global Injury in HI-Treated Brain

Building on the successful optimization of NAM-PNTs in OGD-exposed OWH slices, we further evaluated the efficacy of NAM-PNTs in an *in vivo* hypoxia–ischemia (HI) model in P10 rats. After unilateral left carotid artery ligation and hypoxia exposure to induce unilateral HI, pups were randomized to intraperitoneal (ip) injection of PNTs (sonicated to a length of 124.24 nm, Figure S23; 500 mg/kg), NAM-PNTs (sonicated to a length of 128.16 nm, Figure S23; 25 mg/kg NAM, 500 mg/kg PNTs), free NAM (25 mg/kg), or saline (10 mL/kg) (Figure 4a). PNT and NAM-PNT dosages were derived by performing mass-based scaling from the effective concentration (20 μg/mL) used in *ex vivo* brain slice studies. Preliminary pharmacokinetic (PK) studies confirmed PNT safety, with no mortality or weight loss observed in healthy pups within 72 h of administration, indicating no adverse effects on well-being at this dose (Figure S24). Compared with the saline, free NAM, and PNT groups, animals in the NAM-PNT group lost less weight after surgery and had the highest percentage (7.1%) of weight change by P13 (Figure 4b). The median gross injury scores of all groups were at the maximum score of 4, indicating a high degree of injury; however, lower gross injury was observed with NAM-PNT treatment compared with that of the other groups (Figure 4c).

**Figure 4:**
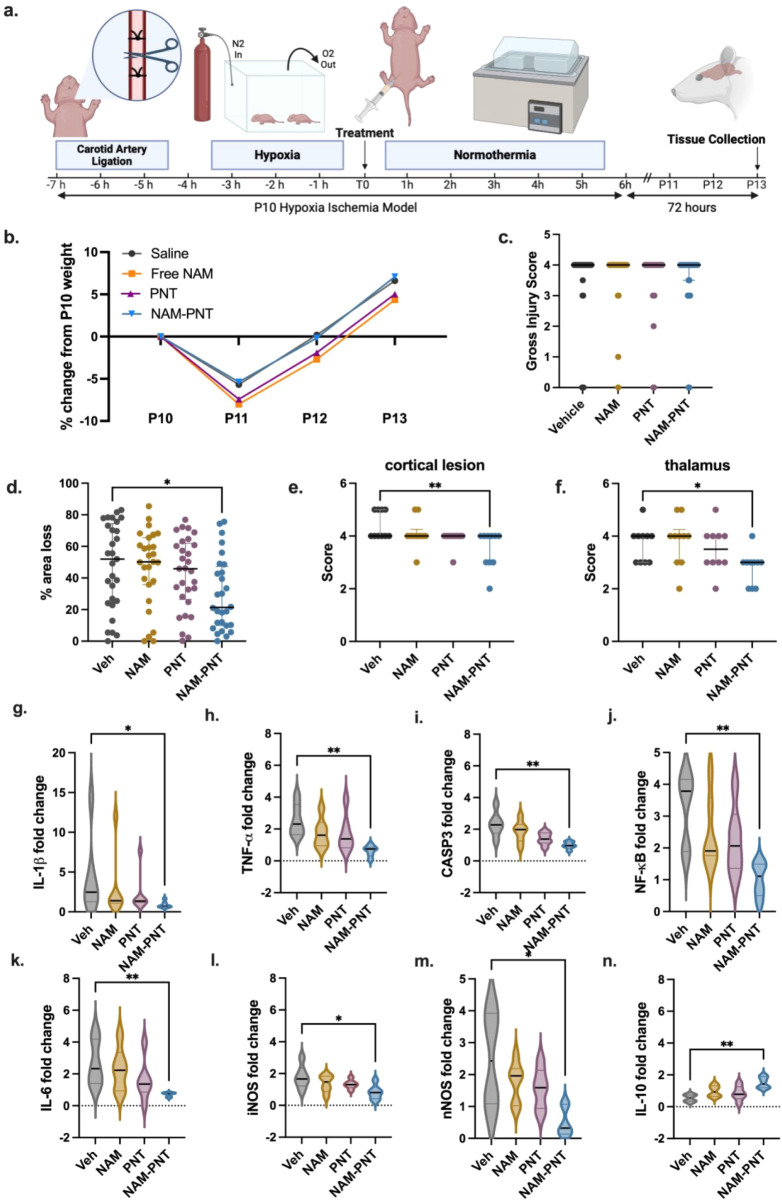
Global brain injury and percent area loss in the HI brain in response to NAM-PNT treatment. a, Schematic diagram of the HI model. Figure created with Biorender.com. b, Weight loss compared to P10 weight after HI. c, Gross injury was assessed on a 0 (least injured) to 4 (most injured) scale for all groups: Vehicle (*n* = 30); free NAM (*n* = 30); PNT (*n* = 31); NAM-PNT (*n* = 31). d, Total area of loss: Vehicle, *n* = 29; free NAM, *n* = 27; PNT, *n* = 29; NAM-PNT, *n* = 28. Statistical significance was calculated via a Kruskal–Wallis test. e, Cortical lesion score 0–5 with NAM-PNT showing significantly lower cortical lesion scoring compared to Vehicle. Statistical significance was calculated via a Kruskal–Wallis test (*n* = 10). f, Thalamus neuropathology data score of 0–5 with NAM-PNT showing significantly lower lesion scoring compared to Vehicle. Statistical significance was calculated via a Kruskal–Wallis test (*n* = 10). For plots c–e, data are presented as scatter dot plots with lines at the median with the interquartile range. g–n, Fold-changes of mRNA markers compared to the contralateral hemisphere showing that NAM-PNT helps regulate RNA expression in P10 rats after HI injury with PNT treatments. Statistical significance was calculated via a Kruskal–Wallis test. Data are presented as violin plots that show the full range with lines at the median with the interquartile range (*n* = 6).

Gross injury scores are only based on the percentage of visual infarct, so further analyses assessing histopathology were conducted. Total area loss was identified by hematoxylin and eosin (H&E) staining and was calculated by taking the ratio of the injured (ipsilateral) hemisphere to the uninjured (contralateral) hemisphere.^64^ The percent area loss was significantly lower in the NAM-PNT group compared to saline (p = 0.03) (Figure 4d, Figure S25). The NAM-PNT group had significantly lower cortical neuropathology compared to the Vehicle group (p = 0.007; Figure 4d) and less injury based on neuropathology in the thalamus (p = 0.047; Figure 4f). The presence of bilateral injury (compared to only unilateral injury) (Figure S26a) and injury in the hippocampus (Figure S26b) were similar across all groups.

RNA was extracted from brain tissue after HI, and cytokine levels in the injured left hemisphere were compared with those in the contralateral hemisphere. Proinflammatory cytokine expression for IL-1 and TNF-α significantly decreased with NAM-PNT administration compared to the Vehicle group (Figure 4g,h). The decreased expression of cell death markers CASP3 and NF-κB was also decreased by NAM-PNT, aligning decreases in apoptosis and inflammatory activation with a reduction in brain area loss (Figure 4i,j). NAM-PNT also significantly reduced elevations in IL-6 (Figure 4k), inducible nitric oxide synthase (iNOS), and neuronal nitric oxide synthase (nNOS) expression in the injured hemisphere compared to the contralateral hemisphere (Figure 4l,m), as well as significantly increased the expression of IL-10 (Figure 4n). These cytokine changes further confirm that NAM-PNTs can reverse the injury responses to HI *in vivo*.

We further explored sex-specific responses to injury and NAM-PNT, as sex can influence therapeutic efficacy in this model,^64^ and previous studies suggest that males may be more susceptible to PARP-1-related cell death.^65^ Females appeared to have higher injury (Figure S27) as has been described previously.^64^ NAM-PNTs reduced injury by approximately 50% compared with the Vehicle group, suggesting similar relative efficacy regardless of sex. Further experiments are likely to be needed to explore the role of timing of NAM-PNT treatment and the number of doses, including repeat dosing over multiple days, to better capture the full therapeutic potential of NAM-PNTs. The detailed mechanism and metabolic fate of NAM delivered via PNTs can also be investigated by analyzing the PK of NAM delivered via PNT and the pharmacodynamics of NAM-PNTs. However, just a single dose of NAM-PNT optimized through our *in vitro* and *ex vivo* models showed significant efficacy *in vivo* in the HI model.

### PNTs are Localized in Microglia in the OGD-Injured Brain and HI-Exposed Animals

To further explore PNT cellular localization, DNS-PNTs were added to OWH slices, and rhodamine-PNT (Rhd-PNT, Figures S28 and S29) was administered i.p. in HI-exposed animals. To mirror the NAM-PNT studies, DNS-PNTs were sonicated for 0, 60, and 120 min and were applied to slices for 24 h. PNT lengths are comparable to those indicated in Table S1. Slices were stained with anti-Iba1 for microglia and anti-NeuN for neurons. In the OWH slices, we observed large agglomerates of PNTs for DNS-PNT0, with limited colocalization in cells (Figure 5a). With increasing sonication time, the reduced length of PNTs led to a more distributed cellular uptake in microglia (Figure 5b). PNTs in the cells were confirmed to be intracellular and not surface-associated with microglia (Figure 5c). PNT uptake in microglia is not surprising given the phagocytic role microglia play in maintaining a homeostatic microenvironment in the brain parenchyma.^66,67^ However, we observed an unexpected morphological change in microglia when they interacted with DNS-PNT0 and DNS-PNT60. There was a significant increase in perimeter and reduction in circularity of the microglia internalizing DNS-PNT0 and DNS-PNT60 compared to DNS-PNT120 (Figure S30). Under healthy conditions, microglia are usually in a surveillance state with a highly ramified phenotype. The bushy morphology with PNT0 and PNT60 may be in response to the internalization of longer PNTs. The internalization of particles with a high aspect ratio can distort the cell shape^68^ and the longer PNTs appear to contort microglia so that they may be internalized. Previous work has shown that PNTs that are hundreds of nanometers in length undergo endocytosis into cells.^30^ Though PNTs actively interact with microglia, the mechanism by which they are internalized remains unclear, although our BV-2 studies suggest that this uptake may be a combination of phagocytosis and caveolae-mediated endocytosis.

**Figure 5:**
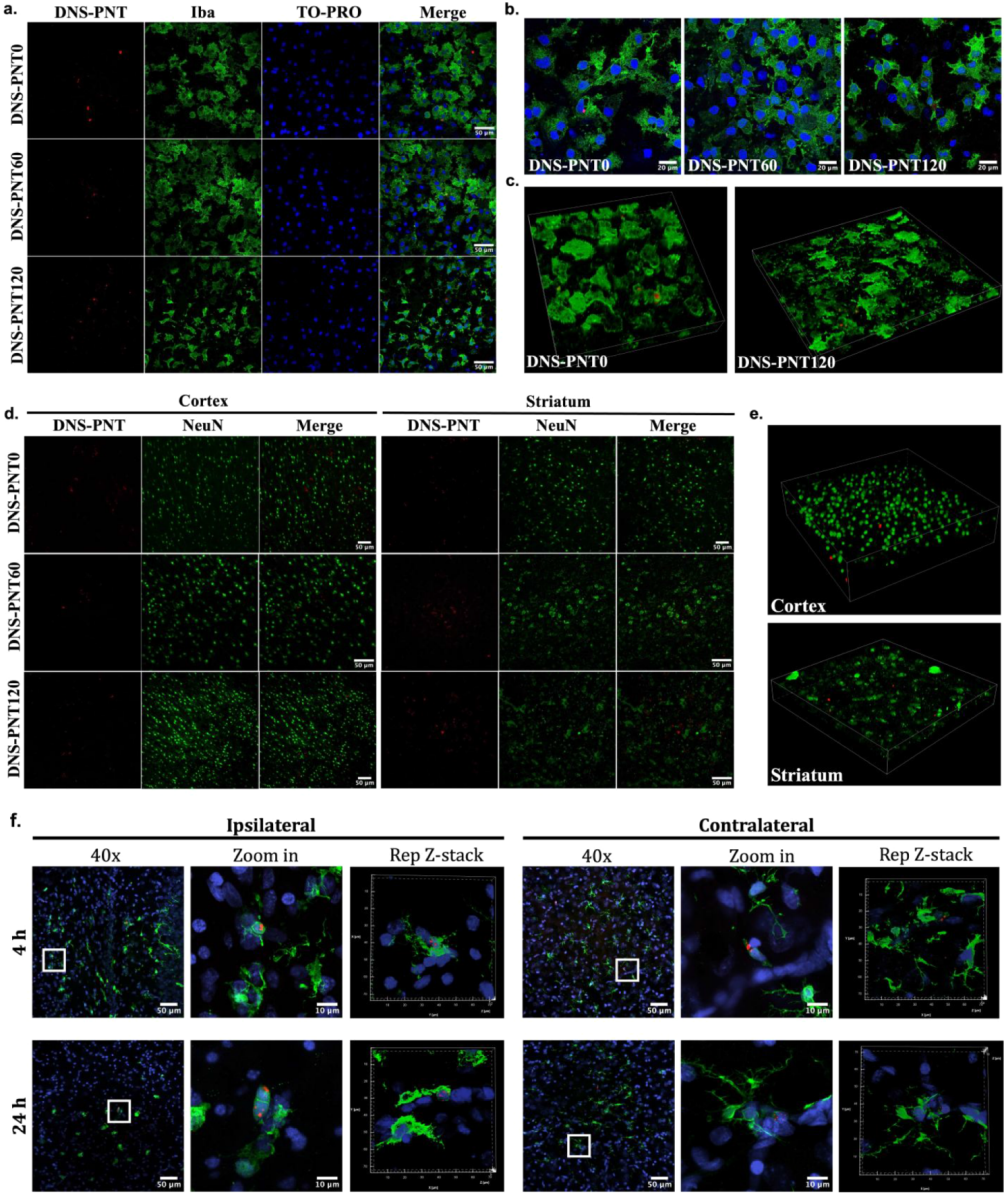
PNT localization in healthy brain slices and HI-exposed brains. a,b, DNS-PNTs (red) localization in Iba1+ microglia (green) and ToPro-3-stained nuclei (blue) in healthy slices with magnifications of 60× (a) and 100× (b). Scale bar: 50 μm. (c) Z-stack images of Iba1+ microglia with DNS-PNT0 and DNS-PNT120. d, DNS-PNTs (red) localization in NeuN+ neurons (green) in healthy slices. Scale bar: 50 μm. e, Z-stack images of NeuN+ neurons with DNS-PNT120 in the cortex and striatum. f. Rhd-PNTs (red) localization in Iba1+ microglia (green) and DAPI-stained nuclei (blue) in unilateral HI-exposed brain slices in the ipsilateral and contralateral hemispheres after 4 and 24 h of injection (40×, 240×, and representative z-stack images).

In OGD-exposed tissue, microglia exhibited a more amoeboid morphology, and PNT colocalization in microglia within OGD slices was reduced compared to healthy OWH slices, although localization was still observed for all three PNT lengths (Figure S31). The morphology of microglia with PNT120 showed more ramified branching resembling that of a surveilling state compared to microglia exposed to PNT0 and 60. This may indicate that the PNTs are altering the phenotype of the microglia, although more analysis of the transcriptomic state of PNT-containing microglia is needed. Neurons showed more limited interactions with PNTs, regardless of length (Figure 5d,e), with z-stack images in both the cortex and striatum showing that DNS-PNTs had no apparent cellular interaction between the PNT and neuronal body (Figure 5e).

PNT tissue-level localization was further explored in the unilateral HI model in P10 rats. PNTs were conjugated with rhodamine B (Rhd-PNT, 200 mg/kg) to enhance signal detection in blood and tissue samples and were administered i.p. for 4 or 24 h. No particles were detected in the i.p. cavity, indicating complete absorption into the systemic circulation. The liver showed the highest Rhd-PNT accumulation at both time points, while lung accumulation was prominent at 4 h but declined by 24 h (Figure S32). Particles were also detected in the kidney at both time points, with much less signal compared to the other organs. Rhd-PNTs were detected in microglia on both the ipsilateral and contralateral sides of the injury, with z-stack imaging confirming their colocalization (Figure 5f). Notably, Rhd-PNTs associated more with amoeboid microglia on the ipsilateral side, suggesting a potential injury-targeting effect.

## Conclusion

In summary, we introduce PNTs that are assembled from sequence-defined synthetic polymers as a viable brain delivery platform for NAM as a NAD+ precursor. PNTs are biocompatible without inducing an inflammatory response in the healthy brain and show localization in microglia. NAM-PNTs improved cell viability, replenished the cellular energy supply and NAD­(H) levels, suppressed inflammation, and reduced cell death after acute brain injury. Our findings highlight the strong therapeutic potential of NAM-PNTs for targeted delivery and energy restoration in the acutely injured neonatal brain, demonstrated through assessments *in vitro, ex vivo,* and *in vivo*. This study marks an innovative application of PNTs in neonatal disease and acute brain injury, paving the way for therapeutic strategies to drive energy regeneration in the brain. NAM-PNTs may also be applicable to other brain diseases where energy depletion is a critical driver of pathology, including other acute traumatic and ischemic brain injuries as well as chronic neurodegenerative conditions such as Parkinson’s and Alzheimer’s disease.

## Methods

### Materials

Solvents were purchased from Fisher and VWR and used without further purification. Millipore ultrapure water was employed throughout the experiments. Rink Amide resin (0.7–1.0 mequiv/g), bromoacetic acid, and hydroxybenzotriazole (HoBt) were purchased from Chem-Impex International, Inc. β-Alanine *tert*-butyl ester (Nce) hydrochloride was purchased from Oakwood Chemical and was deprotected following the previous protocol before use.^36,37^ N,N′-Diisopropylcarbodiimide (DIC), 4-methylpiperidine (PIP), 4-bromophenylamine (Nbrpm), *tert*-butyl-N-(2-aminoethyl)­carbamate (Nae), and trifluoroactic acid (TFA) were purchased from Oakwood Chemical. Nicotinic acid, Fmoc-Gly-OH, and thymine-1-acetic acid were purchased from Sigma-Aldrich. N,N-Diisopropylethylamine, 4-(dimethylamino)­pyridine (DMAP), 1-ethyl-3-(3-(dimethylamino)­propyl)­carbodiimide hydrochloride (EDC), and dansyl chloride (DNS) were obtained from TCI. All amine submonomers were used as received. Nicotinic acid, nicotinamide (NAM), and β-nicotinamide adenine dinucleotide (NAD+) were purchased from Sigma-Aldrich. Other reagents and solvents were used as received, unless otherwise stated.

### Synthesis of Functionalized Peptoids

#### Synthesis of Pep-H

The peptoids were synthesized via solid-phase synthesis, utilizing Rink resin amine as the starting material, by a previously reported method.^36,37^ Specifically, 100 mg of Rink resin amine (0.09 mmol of active NH_2_ groups, 1 equiv) was allowed to swell in N,N-dimethylformamide (DMF) for 10 min. The swollen resins were then filtered, and the Fmoc protecting groups were removed by the addition of 2 mL of a 20% (v/v) PIP/DMF solution. The resulting mixture was shaken at room temperature for 40 min. Following this, the resins were drained and subjected to washing with DMF through five washes of 1 mL each.^69^

Following the deprotection step, the deprotected resins underwent an acylation reaction (Figure 6a).^69^ This involved the use of 1.5 mL of a 0.6 M bromoacetic acid solution and 0.3 mL of a 50/50 (v/v) DIC/DMF mixture. The reaction mixture was shaken for 10 min at room temperature, followed by washing with DMF (5 × 1 mL). Nucleophilic displacement of bromide with the submonomers was accomplished by introducing 1.5 mL of a 0.6 M primary amine solution in *N*-methyl-2-pyrrolidone (NMP) and agitating the mixture for 10 min at room temperature. Subsequently, the solution was filtered, and the resins were washed with DMF (5 × 1 mL). The acylation and displacement reactions, employing appropriate primary amines such as Nbrpm, Nce, or Nae, were iteratively performed until the desired target peptoid sequence (termed as Nbrpm6Nce3Nae3) was achieved (Figure 6b).

**Figure 6:**
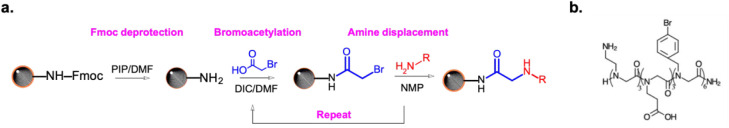
a, General procedure for submonomer synthesis of peptoids. b, Chemical structure of Nbrpm6Nce3Nae3.

#### Synthesis of Pep-NAM

To achieve the NAM-containing peptoid, Nbrpm6Nce3Nae3 (0.09 mmol, 1 equiv) was first allowed to swell in DMF for 10 min following a method published previously.^69^ Briefly, the swollen resins were then filtered, and Fmoc-Gly-OH (268 mg, 0.9 mmol, 10 equiv) and 2 mL of a 50/50 (v/v) DIC/DMF mixture were added. The reaction proceeded by shaking at room temperature overnight. Subsequently, the resins were drained and washed with DMF through five washes (1 mL each). Following this, the Fmoc protecting groups were removed by adding 2 mL of a 20% (v/v) PIP/DMF solution, and the resulting mixture was shaken at room temperature for 40 min.^69^ The resins were then drained and washed with DMF in five washes of 1 mL each. Next, nicotinic acid (111 mg, 0.9 mmol, 10 equiv) and 2 mL of a 50/50 (v/v) DIC/DMF mixture were added, and the reaction was shaken at room temperature overnight. Afterward, the resins were drained and subjected to washing with DMF for five washes of 1 mL each.

#### Synthesis of Pep-Thy and NAD+ Association

To achieve a thymine-containing peptoid, Nbrpm6Nce3Nae3 (0.09 mmol, 1 equiv) was first allowed to swell in DMF for 10 min following a previously published method.^69^ Briefly, the swollen resins were then filtered, and Fmoc-Gly-OH (268 mg, 0.9 mmol, 10 equiv) and 2 mL of a 50/50 (v/v) DIC/DMF mixture were added. The reaction proceeded by shaking at room temperature overnight. Subsequently, the resins were drained and washed with DMF through five washes of 1 mL each. Following this, the Fmoc protecting groups were removed by adding 2 mL of a 20% (v/v) PIP/DMF solution, and the resulting mixture was shaken at room temperature for 40 min. The resins were then drained and washed with DMF through five washes of 1 mL each. Next, thymine-1-acetic acid (166 mg, 0.9 mmol, 10 equiv), EDC (173 mg, 0.9 mmol, 10 equiv), HoBt (122 mg, 0.9 mmol, 10 equiv), DMAP (22 mg, 0.18 mmol, 2 equiv), and 2 mL of DMF were added, and the reaction was shaken at room temperature overnight. Afterward, the resins were drained and subjected to washing with DMF through five washes of 1 mL each. Before NAD+ association, 50 μL of Pep-Thy (1 μM) were sonicated for 60, 120, and 360 min for cutting into different lengths if needed. The resulting PNTs were then added into 950 mL of NAD+ solution (1 mg/mL) and mixed for 30 min. The PNTs were allowed to centrifuge for 15 min at 15,000 g for sample collection. The supernatant was collected for further drug-loading analysis through liquid chromatography with an Eclipse C-18 column (4.6 × 150 mm, 5 μm). The mobile phase was 100% DI with a flow rate of 1 mL/min isocratic. NAD+ standard solutions were used to obtain the calibration curve for each time of the experiment. The drug loading (DL) was identified as follows:

$$\mathrm{Drug}\;\mathrm{loading}\;\left(\mathrm{DL}\right)\;\left(\%\right)=\frac{\mathrm{Starting}\;\mathrm{weight}\;\mathrm{of}\;\mathrm{drug}-\mathrm{weight}\;\mathrm{of}\;\mathrm{free}\;\mathrm{drug}}{\mathrm{Total}\;\mathrm{weight}\;\mathrm{of}\;\mathrm{nanoparticles}}\times 100\%$$

#### Synthesis of Pep-DNS

To achieve the DNS-containing peptoid, Nbrpm6Nce3Nae3 (0.09 mmol, 1 equiv) was first allowed to swell in DMF for 10 min. Next, dansyl chloride (282 mg, 0.9 mmol, 10 equiv) and 150 μL of N,N-diisopropylethylamine were added. The reaction proceeded by shaking at room temperature overnight. Subsequently, the resins were drained and washed with DMF through five washes of 1 mL each.^69^

#### Synthesis of Pep-Rhd

To achieve Rhd-containing peptoid. Nbrpm6Nce3Nae3 (0.09 mmol, 1 equiv) was first allowed to swell in DMF for 10 min. The swollen resins were then filtered, and 2 mL of a DMF solution of rhodamine B (0.9 mmol) and 0.50 mL of 50% (v/v) DIC/DMF were added, followed by agitation overnight at room temperature.^39^ Subsequently, the resins were drained and washed with DMF through five washes of 1 mL each.

#### Purification and Mass Spectrometry Analysis

The detachment of peptoids from the resin beads involved the treatment of bead resins with 3 mL of a 95/5 (v/v) trifluoroacetic acid (TFA)/H_2_O solution for 30 min, accompanied by agitation.^69^ The resulting solution was collected, and the TFA component was evaporated under reduced pressure at 36 °C. Subsequently, the crude peptoid product was dissolved in an 80/20 (v/v) acetonitrile/H_2_O mixture and subjected to purification through reverse-phase high-performance liquid chromatography (HPLC) using a Waters 1525 system equipped with an XBridge Prep C18 OBD column (10 μm, 19 mm × 100 mm).^69^ Purification involved employing a linear gradient of 45–55% acetonitrile in water with 0.1% TFA for Pep-NAM and Pep-THY, or 50–70% acetonitrile for other peptoids. The eluted fractions were characterized by mass spectrometry, following the methodology established in our prior work.^36^

#### Preparation of Peptoid Nanotubes and Characterization

PNTs were prepared using the slow evaporation method to self-assemble the peptoids. Typically, 1 μmol of peptoid was treated with 2 μL of trifluoroacetic acid (TFA) and dried under a flow of N_2_. Subsequently, 100 μL of acetonitrile was added, followed by the addition of 0.5 M NaOH. Deionized water was then added to bring the total volume to 200 μL. The samples were left at 4 °C for slow evaporation until a gel-like solution was obtained. Ex situ Atomic Force Microscopy (AFM) analysis was conducted using a Bruker MultiMode 8 in ScanAsyst mode at room temperature. For sample preparation, 1.5 μL of the self-assembled peptoid was diluted 40 times with deionized water and deposited on a mica substrate. After 5 min, Whatman filter paper was used to remove the solution, and then the dried mica substrate was further dried under N2 flow. The AFM probe consisted of silicon tips on silicon cantilevers (NCHV probes, k = 42 N/m, tip radius). Scanning electron microscopy (SEM) images were captured from the samples deposited on a silicon wafer using the Apreo2S scanning electron microscope by Thermo Fisher. The imaging process employed an ETD detector, an excitation voltage of 2 kV, a current of 0.2 nA, and a working distance of around 8 mm. Powder X-ray diffraction (XRD) data were typically acquired on beamline 8.3.1, which features a 5 T single-pole superbend source with an energy range of 5–17 keV, at the Advanced Light Source located at Lawrence Berkeley National Laboratory.^39^ XRD data were collected by using a 3 × 3 CCD array (ADSC Q315r) detector with a wavelength of 1.11583 Å, and the detector was positioned 200 mm from the sample. For XRD sample preparations, peptoid assembly samples were loaded onto a Kapton mesh (MiTeGen) and dried for XRD measurements.^39^ XRD data were analyzed using custom Python scripts.^39^

#### BV-2 Cell In Vitro Model

Murine microglia BV-2 cells were purchased from ATCC (CRL-2469) and cultured based on previous literature.^70^ Briefly, BV-2 cells were cultured in cell culture media (CCM, high-glucose, phenol red-free DMEM supplemented with 10% FBS, 1% glutamine, and 1% 100 U/mL penicillin–streptomycin) at 37 °C under a 5% CO_2_ atmosphere. The passage number of BV-2 cells used in this study was between 7 and 15. At 70–80% confluency, BV-2 cells were passaged and seeded in a 96-well plate for further experiments.

#### Oxygen–Glucose Deprivation (OGD) on BV-2 Cells

We prepared OGD media as previously described.^52,71^ OGD media consisted of 120 mM sodium chloride (NaCl, MilliporeSigma), 5 mM potassium chloride (KCl, MilliporeSigma), 2 mM calcium chloride (CaCl_2_, MilliporeSigma), 1.25 mM monosodium phosphate anhydrous (NaH_2_PO_4_, MilliporeSigma), 2 mM magnesium sulfate (MgSO_4_, MilliporeSigma), 25 mM sodium bicarbonate (NaHCO_3_, MilliporeSigma), and 20 mM HEPES in DI water.^52^ The OGD medium was sterile-filtered (0.2 μm), titrated to pH 7.4 with 1 M hydrochloric acid (Thermo Fisher Scientific) or 1 M sodium hydroxide (Thermo Fisher Scientific), bubbled with nitrogen gas (Praxair, Danbury, CT, USA) for at least 10 min, and prewarmed to 37 °C prior to use. To initiate OGD, the CCM supernatant was removed, and each well was rinsed once with 100 μL OGD media and then replenished with 100 μL fresh OGD media.^71^ The 96-well plates with the OGD samples were placed in a hypoxia incubator chamber (STEMCELL Technologies, Vancouver, Canada) and placed in a 37 °C incubator. The chamber was flushed with nitrogen gas for at least 10 min and then clamped shut to prevent O_2_ from entering. Cells were incubated in the chamber for another 10 min. Following the OGD, wells were rinsed once with CCM and then replenished with 100 μL of fresh CCM. The cells were then returned to standard culture conditions with the desired treatments added.

#### Cell Cytotoxicity, Viability, and ATP-Level Assays

BV-2 cells were seeded at either 1 × 10^4^ or 2 × 10^4^ per well in a 96-well plate and cultured for 2 days. Cells were treated with free NAM, NAD+, NAM-PNTs, and NAD+-PNTs (sonication times: 0, 60, 120, 240, and 360 min) after OGD for 24, 48, and 72 h. Doses were selected as 2, 20, and 50 μg/mL for PNTs, and the equivalent amount of free NAM and NAD+ was applied (0.11, 1.1, and 2.7 μg/mL). Cell cytotoxicity was identified by the standard 3-(4,5-dimethylthiazol-2-yl)-2,5-diphenyltetrazolium bromide (MTT) assay (Thermo Fisher Scientific) on healthy cells. After 24 h, cells were incubated with fresh media containing 10 μL of 12 mM MTT solution in PBS for 4 h. Following the addition of 50 μL of dimethyl sulfone (DMSO), the absorbance was recorded by a Synergy H1 multimodal microplate reader at a wavelength of 540 nm. Cell viability was measured using the alamarBlue (aBlue) Cell Viability Reagent (Thermo Fisher Scientific). The reagent was diluted 10× in prewarmed CCM. The existing CCM was discarded, and 100 μL of aBlue CCM was added to each well. The cells were returned to the incubator and allowed to incubate for 4 h. The plate was measured directly at 560/590 nm excitation/emission fluorescence on a Synergy H1 multimodal microplate reader. Readings were normalized to those of healthy controls. Cellular ATP levels were tested using a luminescent ATP detection kit (catalog no. ab65348, Abcam) following the manufacturer’s protocol. Data were collected using a Synergy H1 multimodal microplate reader. For the OGD studies, readings were normalized to the average value of the OGD controls.

#### Cell Exocytosis and Uptake Inhibition

BV-2 cells were seeded at a density of 10,000 cells per well in a 96-well plate and cultured for 2 days. Cells were then treated with DNS-PNT (20 μg/mL) in CCM for either 1 or 24 h. Following uptake, the medium containing PNTs was collected, and the cells were further incubated in fresh CCM for 1 or 24 h to allow exocytosis. The supernatant was subsequently collected, and the cells were washed three times with PBS. To quantify the PNT concentration in the medium, a standard fluorescence calibration curve was generated using serial dilutions of PNTs. Fluorescence intensity was measured directly at 335/518 nm excitation and emission using a Synergy H1 multimodal microplate reader. For inhibition studies, BV-2 cells were seeded in 35 mm culture dishes (VWR) at a density of 300,000 cells and cultured for 2 days. Cells were pretreated with inhibitors (10 μM) in CCM for 1 h, followed by incubation with DNS-PNT (20 μg/mL) in CCM for 24 h with or without inhibitors. The inhibitors used were chlorpromazine hydrochloride (Sigma), cytochalasin D (Sigma), amiloride hydrochloride hydrate (Sigma), sucrose (Thermo Fisher Scientific), and genistein (Sigma).^72–75^ After treatment, cells were incubated with LysoTracker (75 nM, Invitrogen) for 1 h and fixed with 10% formalin phosphate buffer (Thermo Fisher Scientific) for 10 min. Subsequently, cells were stained with Tomato Lectin (1 μg/mL, 1 h) and ToPro-3 (0.1 μg/mL, 15 min; Thermo Fisher Scientific) in PBS at room temperature. Confocal images were acquired using a Nikon microscope (40× and 240× oil immersion objectives, 1.30 numerical aperture; Nikon Corporation, Minato City, Tokyo, Japan).

#### Animal Care and Ethical Statement for *Ex Vivo* and *In Vivo* Studies

All animal work was performed in accordance with the recommendations in the Guide for the Care and Use of Laboratory Animals of the National Institutes of Health (NIH). Animals were handled according to the approved Institutional Animal Care and Use Committee (IACUC) protocols (#4383-01, #4383-02, and 4484-01) of the University of Washington, Seattle, WA, USA. The University of Washington has an approved Animal Welfare Assurance (#A3464-01) on file with the NIH Office of Laboratory Animal Welfare (OLAW), is registered with the United States Department of Agriculture (USDA, certificate #91-R-0001), and is accredited by AAALAC International. Every effort was made to minimize suffering. All work was performed using Sprague–Dawley (SD) rats (virus antibody-free CD, *Rattus norvegicus*, IGS, Charles River Laboratories, Raleigh, NC, USA) that arrived at P5–8 with a nursing dam. Dams were housed individually with their litter and allowed to acclimate to their environment. Before removal from the dam at P10, each dam and her pups were housed under standard conditions with an automatic 12 h light/dark cycle, a temperature range of 20–26 °C, and access to standard chow and sterile tap water ad libitum. The pups were checked for health daily.

#### Preparation of OWH Brain Slices and OGD

OWH brain slice preparation and OGD exposure were carried out as previously published.^52,71^ Briefly, healthy male SD rats were injected with an overdose of 100 μL of pentobarbital (Commercial Beuthanasia D, 390 mg/mL pentobarbital, administered at >120–150 mg/kg) intraperitoneally. Once the animal was unresponsive to a toe pinch with tweezers, it was decapitated with sterile surgical scissors. The brain was removed rapidly under aseptic conditions and submerged in ice-cold dissection media consisting of 100% HBSS (Hank’s Balanced Salt Solution, no Mg^2^+, no Ca^2+^, Thermo Fisher Scientific, Waltham, MA, USA), 1% Penicillin–Streptomycin (Thermo Fisher Scientific), and 0.64% w/v glucose (MilliporeSigma, Burlington, MA, USA).^52^ Whole brains were split into hemispheres with a sterile razor blade and sliced coronally into 300 μm-thick sections with a Mcllwain tissue chopper (Ted Pella, Inc., Redding, CA, USA). As previously described, individual slices were separated in ice-cold dissection media using sterile fine-tip paint brushes and transferred onto 30 mm 0.4-μm-pore-sized cell culture inserts (hydrophilic PTFE, MilliporeSigma) before being placed in a nontreated 6-well plate (USA Scientific Inc., Ocala, FL, USA) containing 1 mL of prewarmed (37 °C) slice culture media (SCM; 50% MEM [minimum essential medium, no glutamine, no phenol red, Thermo Fisher Scientific], 21.75% HBSS [with Mg^2+^, with Ca^2+^], 25% horse serum [heat inactivated, New Zealand origin, Thermo Fisher Scientific], 1.25% HEPES [Thermo Fisher Scientific], 1% GlutaMAX Supplement, and 1% Penicillin–Streptomycin).^52,71^ Slices were cultured in a sterile CO_2_ incubator (Thermo Fisher Scientific) at 37 °C with constant humidity, 95% air, and 5% CO_2_. All treatments were applied topically.

#### OWH Sample Preparation for LDH Cytotoxicity

For measuring global cell death, two brain slices were plated per insert. At 4 DIV, 20 μg/mL NAM-PNT0, NAM-PNT60, and NAM-PNT120 in SCM were added to the slices, and the SCM was collected after 24 h of incubation. To generate a positive cell death control, slices were treated for 2 h with 1% TX-100 in SCM. The supernatant was collected following TX-100 treatment. All supernatant samples were immediately stored at −80 °C. Supernatant samples were removed and thawed at room temperature to conduct LDH assays (Cayman Chemical, Ann Arbor, MI, USA).^52,71^ Following the manufacturer’s instructions, 100 μL of LDH reaction buffer was added to 100 μL of sample supernatant in triplicate to 96-well plates on ice, and the plates were transferred to a stir plate in a 37 °C incubator. After 30 min, absorbance at 490 nm was measured on a Synergy H1 multimode microplate reader to detect the production of colorimetric formazan.^71^ All LDH readings were normalized to an acute positive cell death control.

#### Propidium Iodide (PI) Staining

After 24 h of treatment, slices were stained with 1 mL of 5 μg/mL PI (Thermo Fisher Scientific) in SCM for 45 min under standard culture conditions as previously described.^52,71^ The staining solution was placed underneath the inset. Slices were washed twice for 3 min each with sterile 1× PBS at room temperature, followed by a 1 h wash with 37 °C SCM under culturing conditions, and then fixed with 10% formalin phosphate buffer (Thermo Fisher Scientific) for 1 hour. Following two final washes with room temperature 1× PBS, slices were stored covered at 4 °C until they were ready for use. Within 2 weeks, slices were stained for 15 min with 0.1 μg/mL DAPI (Thermo Fisher Scientific) in 1× PBS at room temperature. Slices were washed twice for 3 min each with 1× PBS prior to imaging. Two-channel 40× confocal images (oil immersion, 1.30 numerical aperture, Nikon Corporation, Minato City, Tokyo, Japan) were obtained for PI and DAPI.^52,71^ For every slice, five images were acquired from both the cortex and the striatum. Image acquisition settings were consistent for all images and conditions. For each image, DAPI+ cell nuclei (total cells) and PI+ cell nuclei (dead cells) that were also DAPI+ were counted manually in ImageJ (NIH) after applying an Otsu threshold and fluorescence cutoff to aid in visualization.^52,71^ The fluorescence cutoff was kept consistent across all images. The PI+/DAPI+ cell ratio was expressed as the percentage of dead cells in an individual image.

#### EdU Cell Proliferation Assay and Analysis

For proliferation analysis, the Click-iT Plus EdU Cell Proliferation Kit for Imaging, Alexa Fluor 647 dye (Thermo Fisher Scientific), was used as previously described.^52^ Briefly, EdU solution was added to live brain slices after OGD via culture media at 20 μM for 16 h. Following fixation, an azide-conjugated dye was reacted with EdU-labeled cells in the presence of a copper catalyst for 1 h. Following a 1× PBS wash step, cellular nuclei were stained with a 0.1 μg/mL solution of DAPI in 1× PBS for 15 min prior to imaging. Three images were acquired at 40× magnification using 405 and 647 nm laser lines in the cortex and striatum. Each experimental group contained 3 brain slices, and image acquisition settings were kept constant throughout. An ImageJ macro was used to set consistent LUT values prior to the manual counting of total cells positive for DAPI and total cells positive for EdU in the acquired images. The ratio of these values (EdU/DAPI) represents the percentage of proliferating cells.

#### Total NAD Level and NAD+/NADH Ratio Analysis

OWH slices were treated with or without OGD at 4 DIV and treated with 20 μg/mL NAM-PNT0 and NAM-PNT120 for 24 h. Subsequently, the slices were harvested from the membrane, washed with PBS, and lysed using 400 μL of lysis buffer. The total NAD and NAD+/NADH ratio were assessed by the NAD+/NADH Quantification Kit (MAK037, Sigma-Aldrich) according to the manufacturer’s instructions.^76^

#### Immunofluorescence Staining and Imaging

Brain slices were prepared, and formalin was fixed for at least an hour. Slices were then incubated with primary antibodies for Olig2 (oligodendrocytes), NeuN (neurons), or Iba1 (microglia). On fixed slices, AlexaFluor 488 Anti-NeuN (rabbit anti-NeuN, Abcam, cat. no. ab190565, Cambridge, UK) at a 1:500 dilution in 1× PBS containing 1% Triton X-100 was applied overnight at 4 °C. Following a 1× PBS wash step, slices were stored at 4 °C until imaged. Iba1 (anti-Iba1, rabbit, FUJIFILM Wako Pure Chemical Corporation) was stained at a 1:250 dilution, and Olig2 (Anti-Olig2 Antibody, MilliporeSigma, Cat#MABN50) stained slices were stained at a 1:500 dilution in 1× PBS containing 1% Triton X-100 and 3% goat serum (MilliporeSigma, Cat#S30-100 mL) overnight at 4 °C. After washing with 1× PBS, Iba1 and Olig2 slices were stained with an AlexaFluor 488 goat antirabbit secondary antibody (ThermoFisher Scientific, Cat#A11034) and AlexaFluor 555 goat antimouse secondary antibody (ThermoFisher Scientific, Cat#A28180) at a 1:500 dilution, respectively, in 1× PBS containing 1% Triton X-100 for 2 h. An antipoly-ADP-ribose binding reagent (MilliporeSigma, cat. no. MABE1031) followed the same staining protocol as Iba1 at a 1:1000 dilution. Either DAPI or ToPro-3 (Thermo Fisher Scientific) was stained at a concentration of 0.1 μg/mL in 1× PBS for 30 min at room temperature.

#### DNS-PNT Localization in Microglia and Neurons

After 4 DIV, 20 μg/mL DNS-PNT0, PNT60, and PNT120 were added to the slices for 24 h of incubation. The slices were either stained with AlexaFluor 488 Anti-NeuN or Iba1 (with an AlexaFluor 488 goat antirabbit secondary antibody). Two-channel 40× and 60× confocal images (oil immersion, 1.30 numerical aperture, Nikon Corp., Minato City, Tokyo, Japan) were taken to assess PNT localization. Image acquisition settings were consistent across all images and conditions.

#### Reverse Transcriptase Quantitative Polymerase Chain Reaction (RT-qPCR)

Changes in proinflammatory, anti-inflammatory, cell death, and oxidative stress gene expression profiles following PNT treatment were quantified using RT-qPCR, adapted from previously published methods.^77^ Briefly, for each condition, three brain slices were cultured together on the same membrane. Slices were preserved in RNALater (Thermo Fisher, Waltham, MA, USA) and kept at 4 °C prior to processing to prevent RNA degradation.^77^ The RNA from homogenized brain slices was extracted with TRIzol reagent, pelleted at 15,000 × *g*, and washed several times with ultrapure DEPC water (Thermo Fisher, Waltham, MA, USA) and cold 70% ethanol, and the RNA final concentration was measured using a NanoDrop. cDNA was diluted to 20 ng/μL with ultrapure RNA-free water.^77^ RNA was transcribed into cDNA using a Thermo Fisher (Waltham, MA, USA) Reverse Transcription RNA to cDNA kit. qPCR was run using the transcribed cDNA and Bio-Rad (Hercules, CA, USA) SYBR Green Master Mix. Primers included anti- or proinflammatory cytokines (IL-1β, IL-6, IL-10, NF-κB), inflammatory cytokine (TNF-α), and a housekeeping gene (GAPDH) (Table S2). The qPCR ran at 95 °C for 30 s, 95 °C for 5 s, and then 55 °C for 45 s for 40 cycles.^77^ The gene expression changes in the OGD-conditioned and PNT treatment groups were normalized to the healthy controls to quantify fold-expression change. Results were statistically analyzed with one-way ANOVA (Kruskal–Wallis multiple comparisons tests) and normalized by the median Cq value for all samples.^77^

#### Unilateral HI Brain Injury

On P10, pups were separated from their dams, weighed, sexed, and randomized into experimental groups. Buprenorphine (SQ, 0.05 mg/kg) was given to all pups at least 30 min prior to surgery. Anesthesia with isoflurane (3% for induction, 1.5–2.0% for maintenance) was blended with 100% O_2_ via a nose cone under a dissecting microscope. A mixture of bupivacaine and lidocaine (1.5 mg/kg) was injected intradermally along the incision site just prior to surgery. Under a dissecting microscope, a small (5 mm) midline-neck incision was made. The left common carotid artery was identified, ligated twice with 6–0 surgical silk, and transected between the ligations. The incision was closed with tissue adhesive (Vetbond, 3M, Minnesota, USA). The pups were maintained in a temperature-controlled water bath before and after unilateral ligation of the left carotid artery to maintain normothermia. After all the animals recovered from anesthesia, they were returned to the dams for a minimum of 1 h before placement in a hypoxic chamber in a temperature-controlled water bath.^78,79^ Briefly, the animals were sealed in the hypoxia chamber, and 8% of the O_2_ (92% N_2_) was administered at a rate of 2.5 L/min. During hypoxia, the temperature of the water bath was adjusted to maintain a target rectal temperature, measured via a rectal probe, of two sentinel animals at 34–35 °C. When the oxygen within the chamber reached 8%, hypoxia was maintained for up to 180 min or 20% mortality, whichever occurred first. A research scientist continuously monitored the pups for respiration and decreased movement for the entire hypoxia duration. After the hypoxia period, the pups were given an additional 30 min rest period with the dams.^78,79^ For all experiments, the temperature of the pups was measured immediately after the 30 min rest period. 30 min after hypoxia, the dose of PNTs (500 mg/kg), saline (Vehicle, 10 mL/kg), or a free drug (25 mg/kg) was administered, and the animals were then continuously monitored for 5 h in a water bath adjusted to maintain the animals at 37 °C rectal temperature. This normothermia is critical to preventing hypothermia caused by the injury and avoiding any changes in early thermoregulation that may confound the association between treatment and outcome.^80^ The animals were then returned to the dam. Animals were monitored and weighed 1–2 times daily and checked for their general appearance (i.e., dehydration, abnormal posture, weight loss).

#### Immunohistochemistry

To assess tissue-level biodistribution, the ipsilateral and contralateral hemispheres of the brain, a portion of the lung and liver, and one kidney were fixed in a formalin-to-30% sucrose gradient and sliced into 30 μm slices using a Leica cryostat based on previously described methods.^77,81^ For brain distribution analysis, tissue sections were incubated with primary antibodies for microglia (1:250 rabbit anti-Iba1, Wako, Fujifilm, Minato City, Tokyo, Japan) in 1× PBS containing 1% Triton X-100 (Sigma-Aldrich) and 3% normal goat serum (Sigma-Aldrich).^77^ Sections were incubated with primary antibody solutions for 4–6 h at room temperature in a humidified dark chamber, followed by two washes in 1× PBS. Secondary antibodies, prepared in 1× PBS containing 1% Triton X-100, were applied to the tissue slides for 2 h, after which the slices were washed twice in 1× PBS and then stained with DAPI (1:10,000; Thermo Fisher Scientific). Slides were washed, air-dried for 30 min in the dark, and mounted with Dako mounting medium (Agilent Technologies, Santa Clara, CA, USA) under glass coverslips. Samples were stored at 4 °C until imaging with an A1 confocal microscope (Nikon, Tokyo, Japan) and at −20 °C for long-term storage. For other organs (lung, liver, and kidney), tissue sections were stained with DAPI and imaged using confocal microscopy.

#### Gross Injury and Area Loss

Gross injury and area loss were measured based on methods previously described.^78,82^ Briefly, at 72 h after HI, animals received an overdose of pentobarbital before transcardiac perfusion with 1× PBS followed by 10% neutral-buffered formalin. Immediately following brain extraction, a photo of each whole brain was taken and subsequently analyzed by an individual who was blinded to group allocation.^78^ As previously described, gross brain injury in the hemisphere ipsilateral to ligation was assessed on a five-point ordinal scale (0–4) as follows: 0 = no injury, 1 = mild injury with <25% lesion of the ipsilateral hemisphere, 2 = 25%–50% lesion, 3 = 51%–75% lesion, and 4 = ≥75% injury.^78^ Whole brains were postfixed in 10% neutral-buffered formalin for at least 48 h. Following the fixation, blocks of brain were obtained. Using external landmarks, brains were cut at approximately the level of the striatum (block 1) and at the level of the hippocampus and thalamus (block 2). The tissue samples were paraffin-embedded, cut into 5-μm sections, and stained with hematoxylin and eosin (H&E). For area loss, briefly, two 5 μm sections from the slices best representing the cortex, hippocampus, basal ganglia, and thalamus were selected.^78,79^ Virtual slides were exported as 600 dpi images. The optical density and hemispheric area of each section were analyzed with ImageJ software (National Institutes of Health, Bethesda, MD, USA) by another blinded individual. The average percentage area loss from the two sections (one at the level of the frontal cortex and the other at the mid-hippocampal level) was calculated using the following formula: [1 – (ipsilateral area/contralateral area)] × 100.^78,79^

#### Histopathological Evaluation

H&E-stained slides from saline (n = 20), free NAM (n = 18), BPNT (n = 18), and NAM-PNT (n = 18)-treated animals were evaluated by a board-certified veterinary pathologist blinded to the group assignment of the rats. A previously reported nine-step scoring system for HIE^83^ was employed, with modifications, to grade the following regions: cerebral cortex, thalamus/midbrain, and hippocampus. Lesions in the cortex were scored semiquantitatively using a 0–5 scale, where “0” indicated no detectable lesion; “1” indicated small focal or multifocal area(s) of neuronal cell loss without white matter cavitation, comprising 10% of the cortex unilaterally or <5% bilaterally (3 foci or fewer), “2” indicated multifocal to coalescing lesion damage with neuronal cell loss and/or white matter cavitation, affecting 10–40% of the cortex (unilateral) through all levels of the cortex without cavitation; “3” indicated multifocal to coalescing lesions affecting 40–75% of the cortex unilaterally or up to 33% bilaterally with or without cavitation, “4” indicated severe lesions with cavitation affecting >75% of the cortex unilaterally or >50% bilaterally; and “5” indicated very severe lesions with >90% neuronal loss and extensive cavitation/collapse of the cortex or striatum. Lesions of the hippocampus were scored using a 0–5 scale, where “0” indicated no detectable lesion, “1” indicated rare necrotic neurons with <5% of the hippocampus affected unilaterally; “2” indicated multifocal mild necrotic neurons, 6–25% unilaterally or up to 10% bilaterally affected, “3” indicated multifocal to coalescing necrotic neurons, 25–50% unilaterally or 10–25% bilaterally affected, “4” indicated 50–75% of neurons necrotic unilaterally or >25% bilaterally; and “5” indicated >75% of neurons necrotic. Lesions of the thalamus/midbrain were scored using a 0–5 scale, where “0” indicated no detectable lesion; “1” indicated small focal areas to ≤3 small areas of neuronal cell loss/necrotic neurons; “2” indicated multifocal mild to coalescing lesions, <10%, with no parenchymal loss; “3” indicated 10–25% of the thalamus/midbrain affected; “4” indicated 25–50% of the thalamus/midbrain affected with parenchymal loss/collapse; and “5” indicated >50% affected. In addition, an extra point was given to brains with bilateral injury.^78,79^

Scores from each region were summed to yield the final score, ranging from 0 to 16. For figures, the median score from each group was calculated, and an animal representing that median score (or within 1 point of the median score) was used to show pathology in the various regions of the brain.^79^ Images of lesions captured from the digitally scanned slides were exported and plated with Adobe Photoshop Elements. Image brightness and contrast were adjusted using White Balance levels and/or Auto Contrast manipulations applied to the entire image. Original magnification and scale bars are stated.

### Statistical Analysis

Statistical analysis was performed in GraphPad Prism version 10.0.3 (GraphPad Software, San Diego, CA, USA). Graphs for the cell features compared to the control and treatment groups are displayed as the median with interquartile range, and all data points are shown. Therapeutic results of free NAM, NAD+, NAM-PNT, and NAD+-PNT in cells and slices were compared using the Kruskal–Wallis test, except for cell viability at different doses and the proliferation study in slices that used ordinary two-way ANOVA. All p-values <0.05 were considered statistically significant.

## Supplementary Material



## Notes

Detailed chemical characterization of the synthesized peptoids (Figures S1–S4), AFM and SEM images of PNT structure (Figures S5–S7), additional PNT length distribution data (Figure S8), drug-PNT characterization (Figure S9), BV-2 study controls and free drug dosing studies (Figures S10–S11), BV-2 OGD exposure response (Figure S12), metabolic activity response to free drug and PNTs in healthy and OGD-exposed BV-2 cells as a function of PNT dose and length (Figures S13–S15), intracellular ATP level changes in response to PNT treatment and OGD (Figure S16), PNT exocytosis and inhibition of endocytosis quantification data in BV-2 cells (Figures S17–S18), representative cell death and cell proliferation images in OGD-exposed and PNT-treated OWH brain slices (Figures S19–S20), mRNA expression fold changes in response to PNT treatment on healthy OWH slices (Figure S21), representative images of PAR signal in response to PNT treatment of OGD-exposed OWH slices (Figure S22), characterization data of PNTs used in *in vivo* studies (Figure S23), weight change and representative pathology images from the PNT treatment study in HI-exposed rats (Figures S24–S25), additional pathological scoring analysis for PNT-treated HI-exposed rats (Figure S26), sex-based differences in total area loss in HI-exposed rats treated with PNTs (Figure S27), additional characterization of Rh-labeled PNTs used *in vivo* (Figures S28–S29), microglia morphological analysis in PNT-treated HI-exposed rat brains (Figure S30), and representative PNT localization images in the brain and organs in rats (Figures S31–S32); quantification of PNT length as a function of sonication time (Table S1) and primers used for RNA analysis (Table S2) (PDF)
