# Supplementary material for: Nicotinamide-Loaded Peptoid Nanotubes for Energy Regeneration in Acute Brain Injury
Source: ACS Nano. 2026 Apr 13;20(16):12250–67. doi: 10.1021/acsnano.5c19897 (PMC13131050; doi:10.1021/acsnano.5c19897)
Supplement: Supplementary file 1 [file nn5c19897_si_001.pdf]

Supplemental Information

Nicotinamide-loaded Peptoid Nanotubes for Energy Regeneration in Acute Brain Injury

Hui Du[1], Thi Kim Hoang Trinh[2], Olivia C. Brandon[3], Renyu Zheng[1,2], Haoyu Wang[1], Kylie Corry[3], Thomas R. Wood[3], Chun-Long Chen[1,2], and Elizabeth Nance[1,4,5]*

[1]Department of Chemical Engineering, University of Washington, Seattle WA 98195, USA
[2]Physical Sciences Division, Pacific Northwest National Laboratory, Richland, WA 99352, USA.
[3]Division of Neonatology, Department of Pediatrics, University of Washington, Seattle, WA 98195, USA
[4]Department of Bioengineering, University of Washington, Seattle, WA 98195, USA
[5]Department of Radiology, University of Washington, Seattle, WA 98195, USA
*Corresponding author: eanance@uw.edu

a)

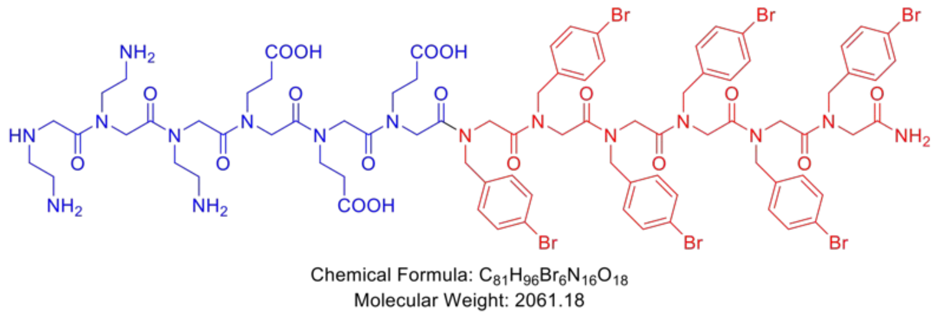

b)

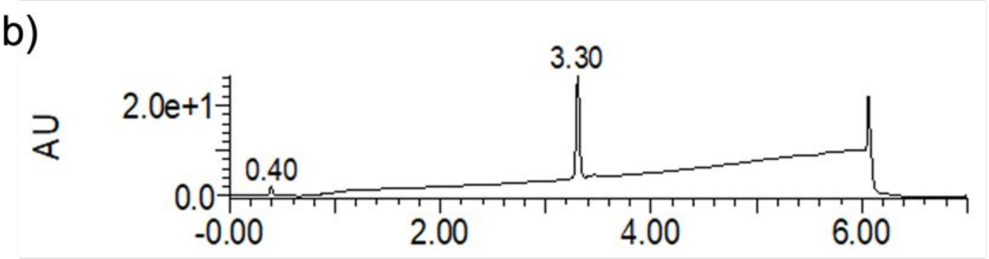

c)

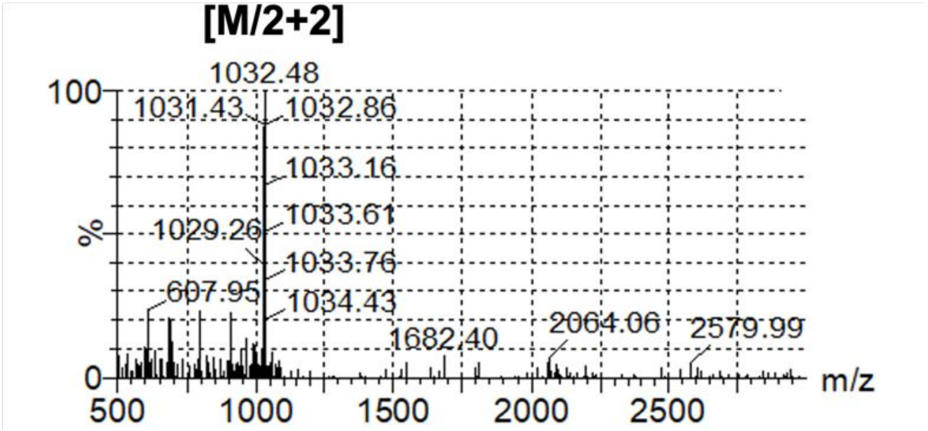

Figure S1. LC-MS analysis of PNT. a) Chemical structure of PNT. b) LC profile of PNT run under gradient of acetonitrile from 5 -95 %. c) ESI mass analysis of PNT.

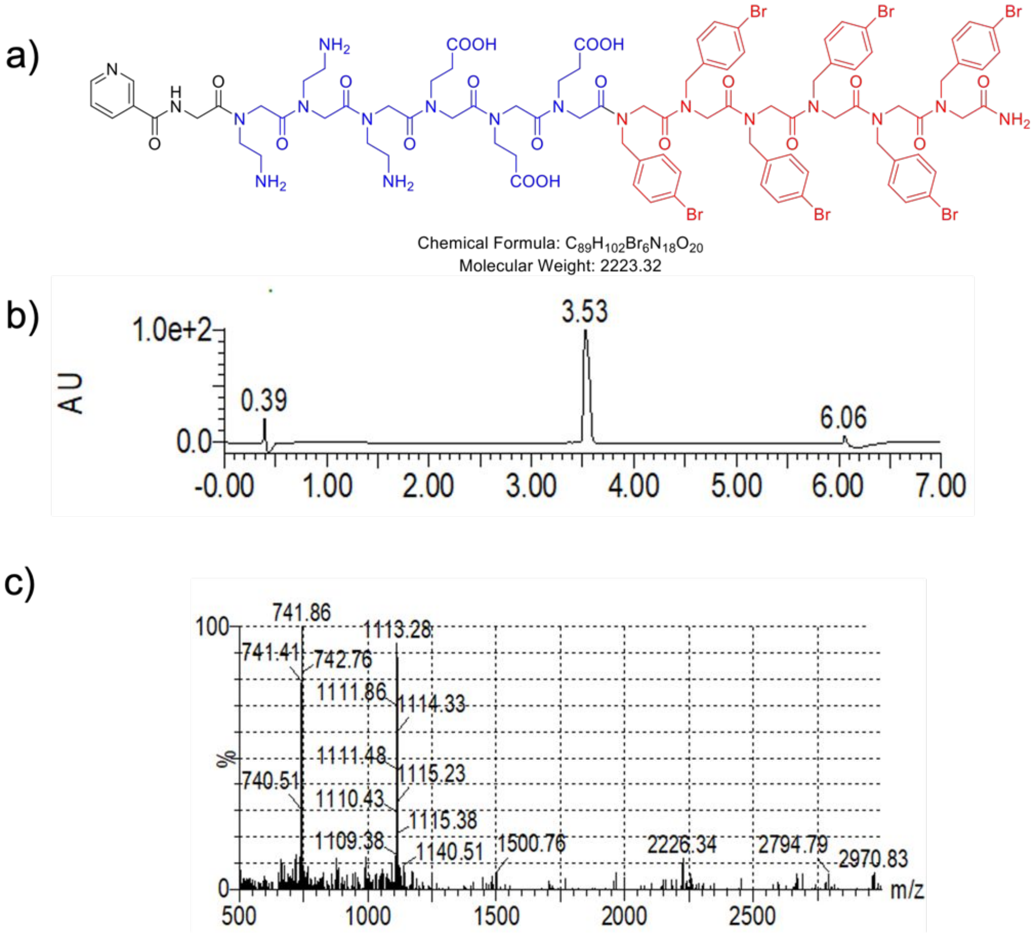

Figure S2. LC-MS analysis of NAM-PNT. a) Chemical structure of NAM-PNT. b) LC profile of NAM-PNT run under gradient of acetonitrile from 5 -95 %. c) ESI mass analysis of NAM-PNT.

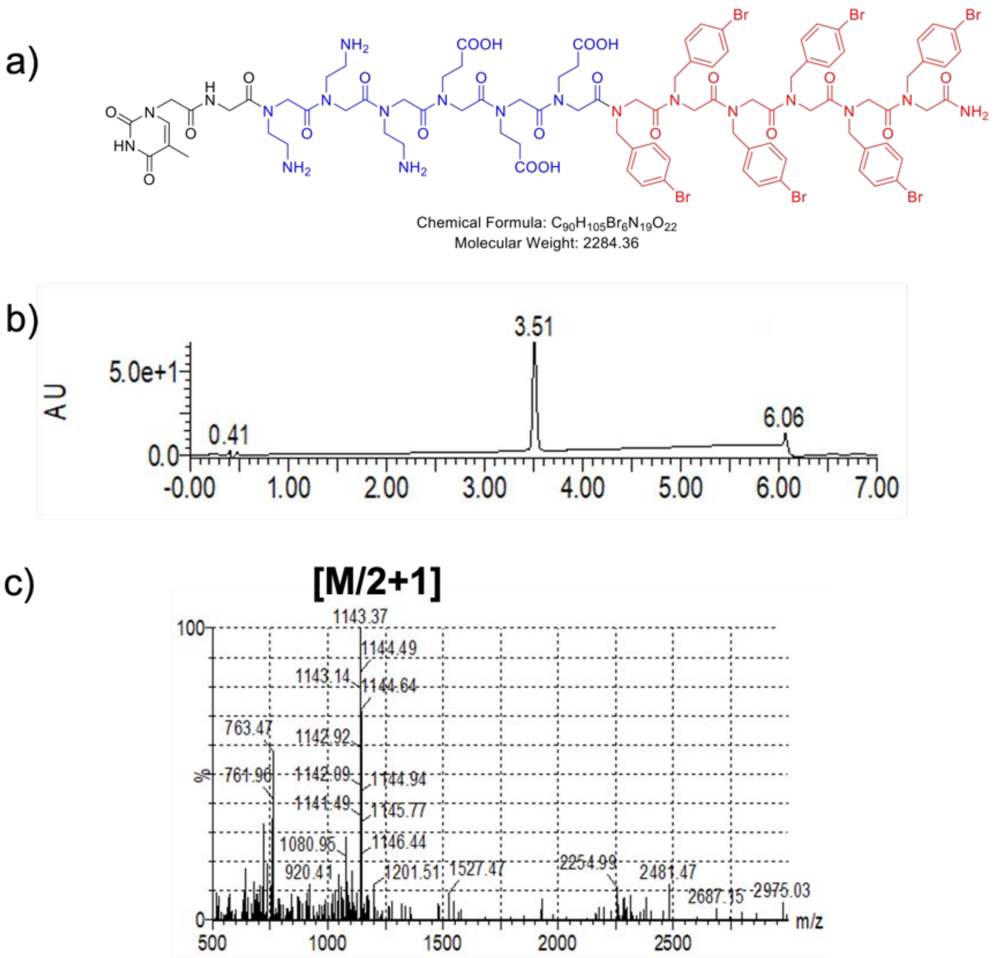

Figure S3. LC-MS analysis of Thy-PNT. a) Chemical structure of Thy-PNT. b) LC profile of Thy-PNT run under gradient of acetonitrile from 5 -95 %. c) ESI mass analysis of Thy-PNT.

d)

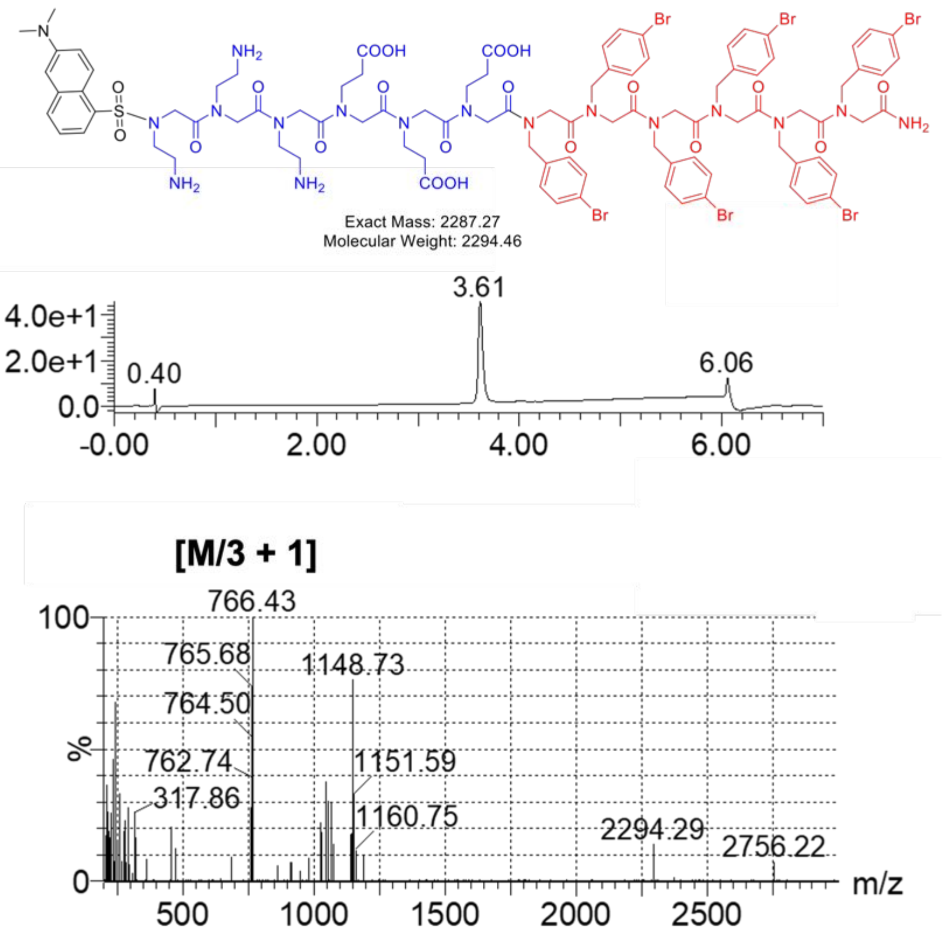

Figure S4. LC-MS analysis of DNS-PNT. a) Chemical structure of DNS-PNT. b) LC profile of DNS-PNT run under gradient of acetonitrile from 5 -95 %. c) ESI mass analysis of DNS-PNT.

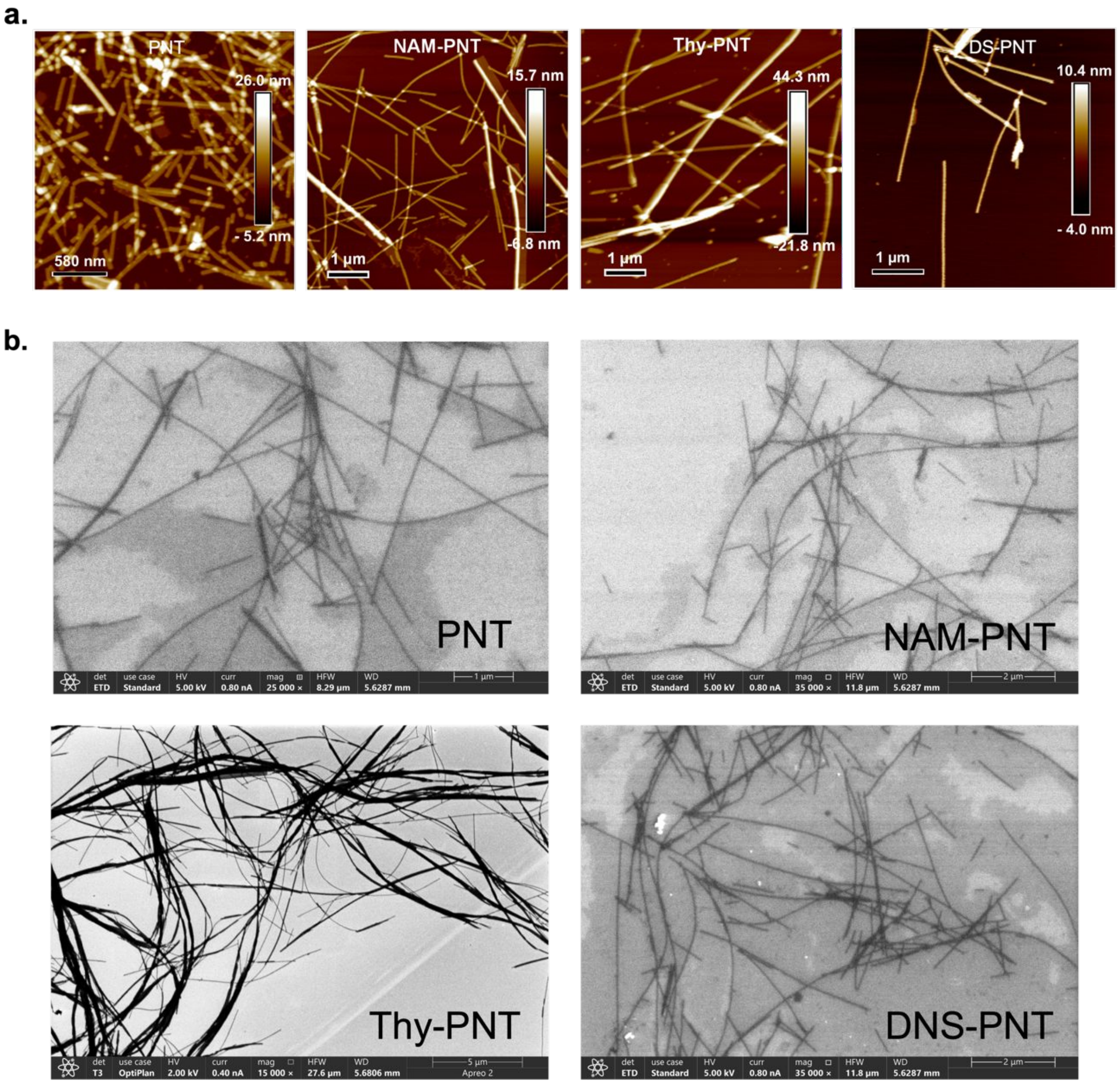

Figure S5. a) Representative AFM images of PNT, NAM-PNT, Thy-PNT, and DNS-PNT, respectively. b) Representative SEM images of PNT, NAM-PNT, Thy-PNT and DNS-PNT.

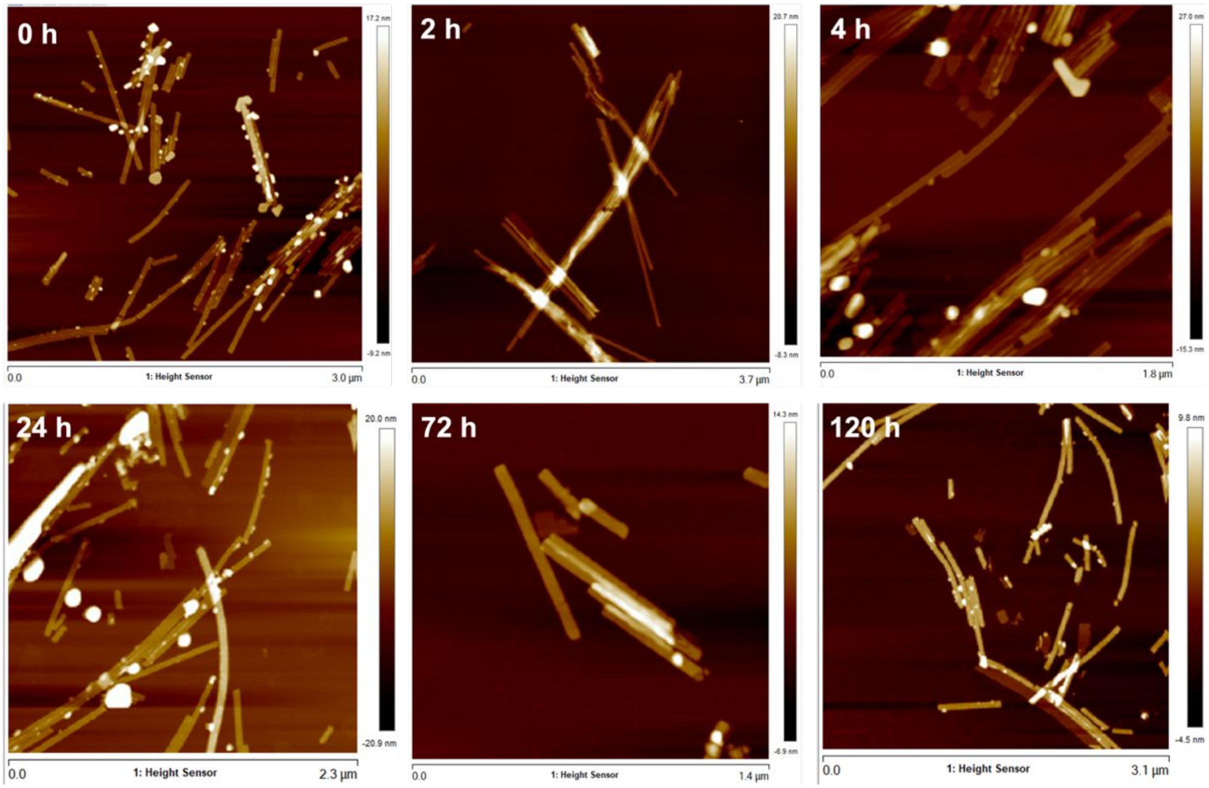

Figure S6. AFM images show the structure of NAM-PNTs in PBS incubated for up to 120 hours.

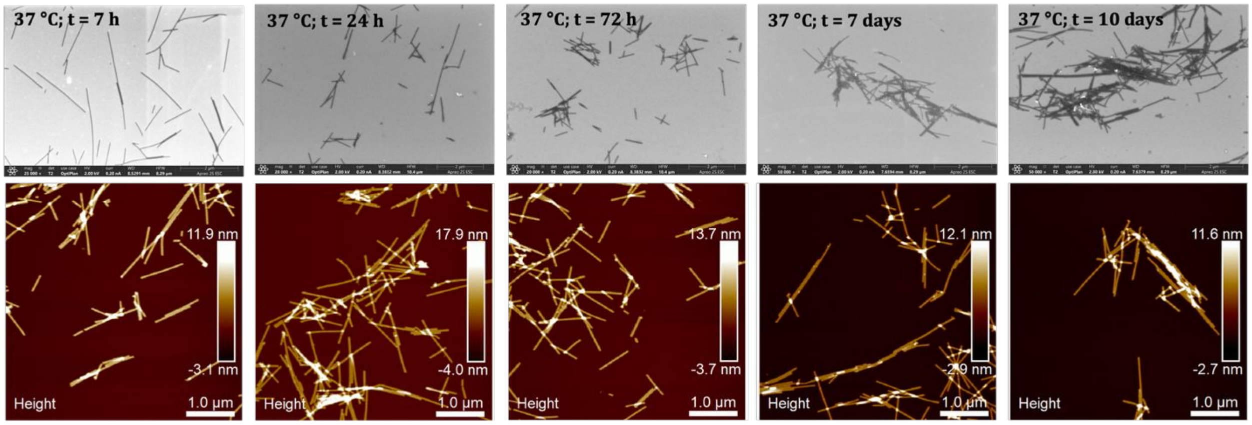

Figure S7. Representative images of NAM-PNTs obtained using scanning electron microscopy (SEM, top) and atomic force microscopy (AFM, bottom), showcasing their structural integrity after incubation in 0.2 wt% pronase in PBS at 37°C for varying durations.

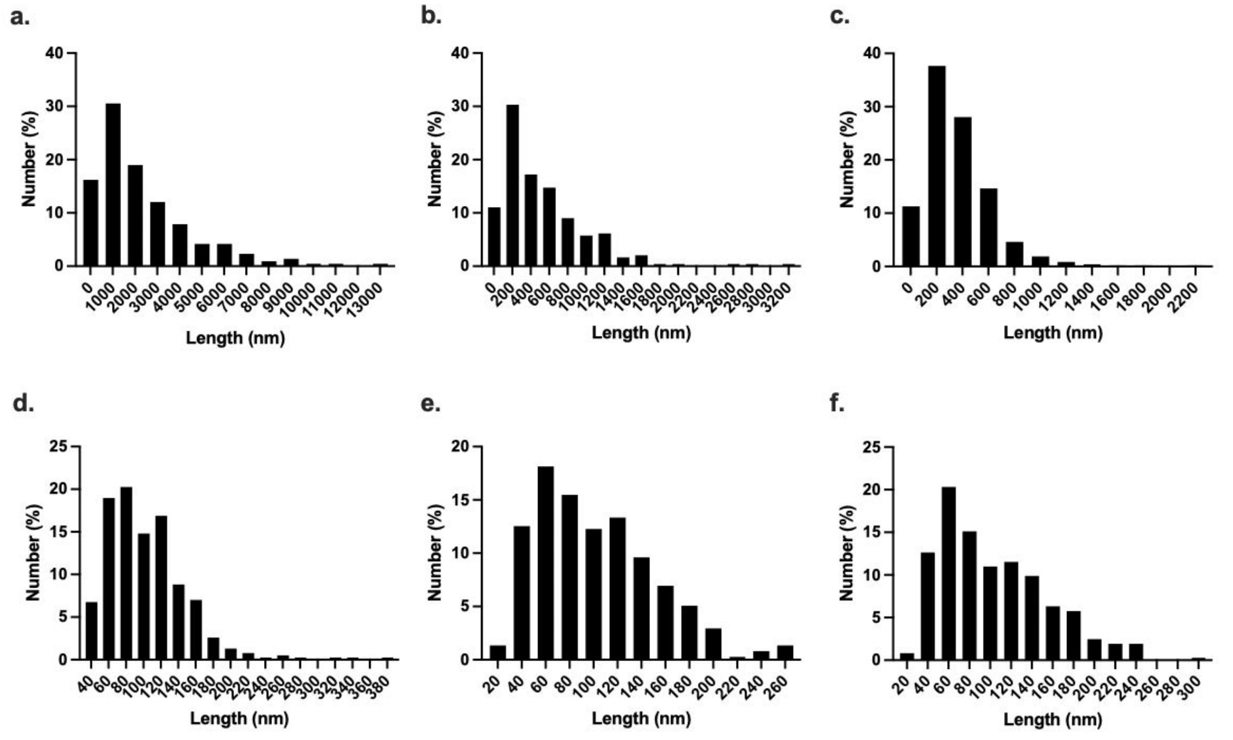

Figure S8. PNT length frequency distribution from 0 to 480 minutes sonication.

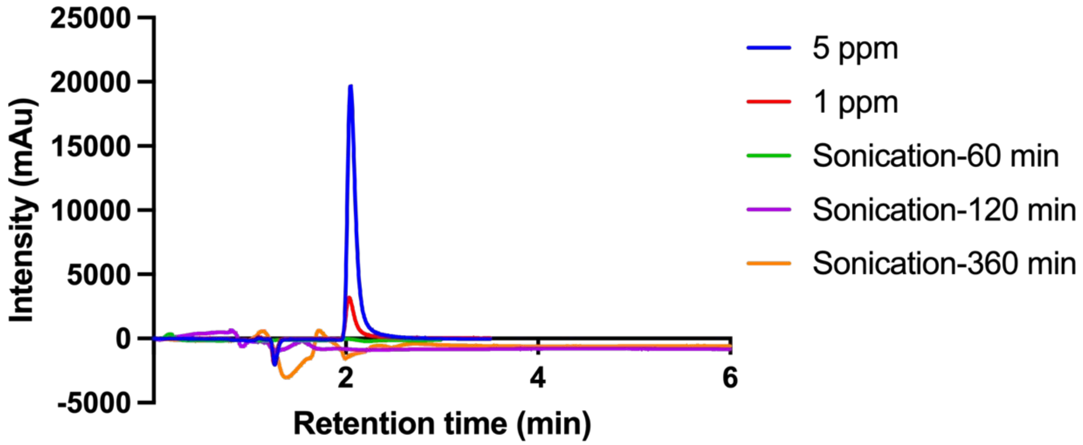

Figure S9. Liquid chromatography of NAM concentration in the NAM-PNT supernatant up to 360 min of sonication. NAM was measured via liquid chromatography. 5 ppm and 1 ppm NAM solutions serve as standards for comparison.

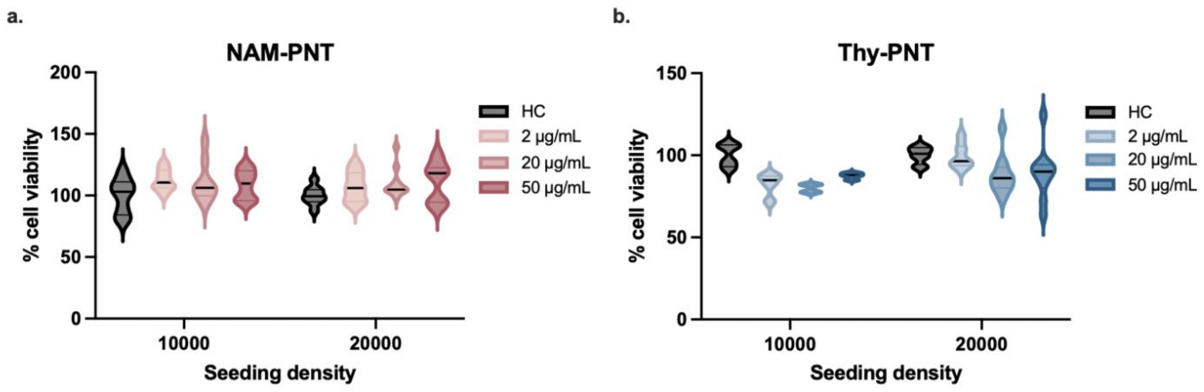

Figure S10. Seeding population effect on cell viability for a) NAM-PNT and b) Thy-PNT. Data are presented as violin plots that show the full range with lines at the median with interquartile range. (n=8-10).

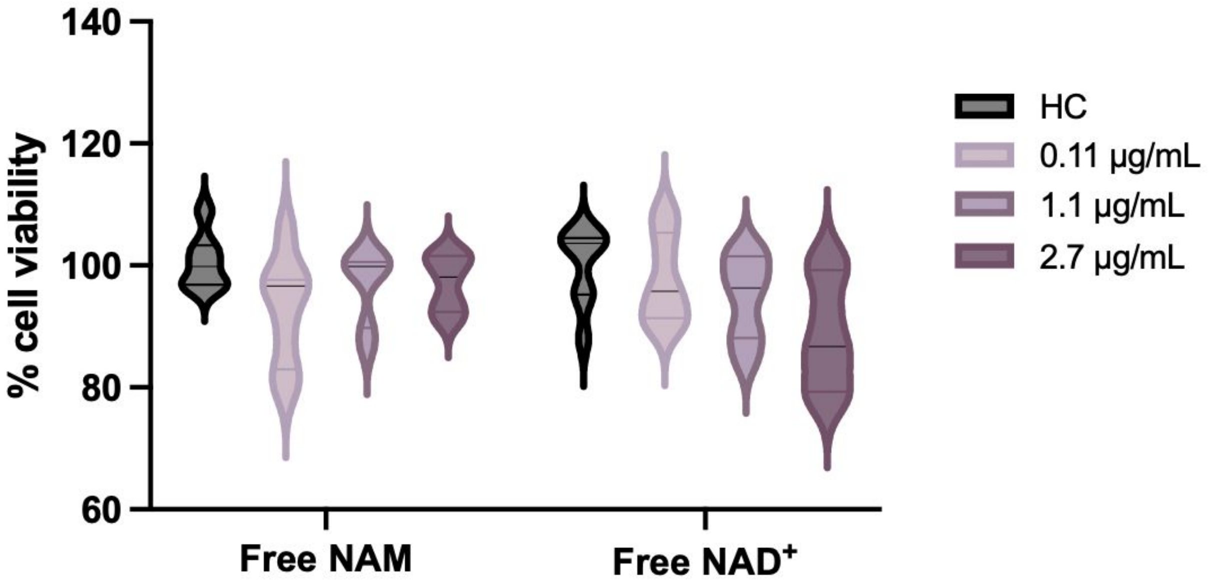

Figure S11. MTT data showing healthy BV-2 cells (HC) treated with 3 doses of free NAM or free NAD+ for 24 h (dose equivalent to NAM-PNT). Data are presented as violin plots that show the full range with lines at the median with interquartile range. (n=7-8).

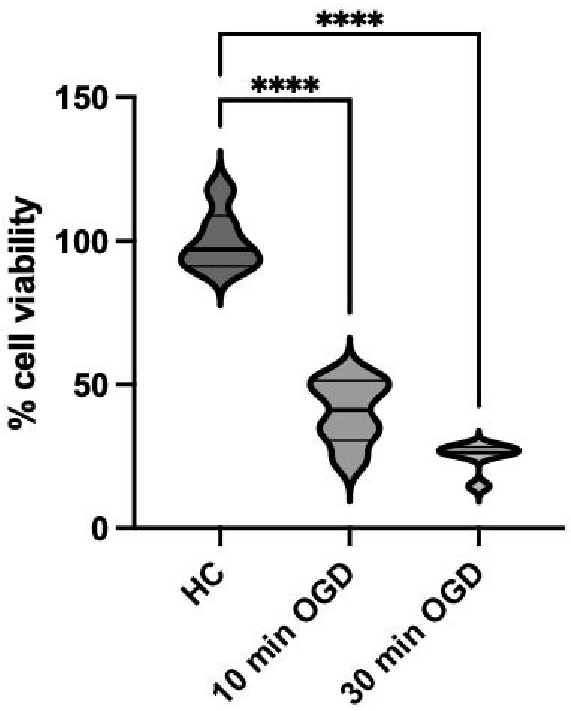

Figure S12. Difference in cell viability, as measured by MTT assay, after 10 min and 30 min of OGD compared to the healthy control (HC). Statistical significance was calculated via a Kruskal-Wallis test. Data are presented as violin plots that show the full range with lines at the median with interquartile range. (n = 6).

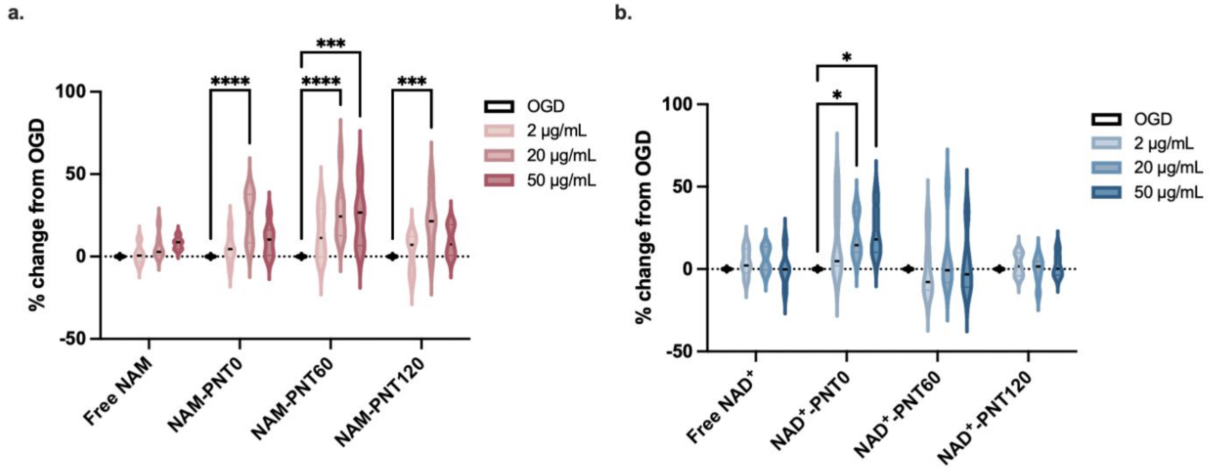

Figure S13. Change in metabolic activity of a) free NAM, and three lengths of NAM-PNTs, and b) free NAD+ and three lengths of NAD+-PNTs compared to the non-treated OGD cells. A dose-dependent effect was also observed, where 20 μg/mL NAM-PNTs led to the highest % change from OGD. Statistical significance was calculated via ordinary two-way ANOVA. Data are presented as violin plots that show the full range with lines at the median with interquartile range. (n=5-8).

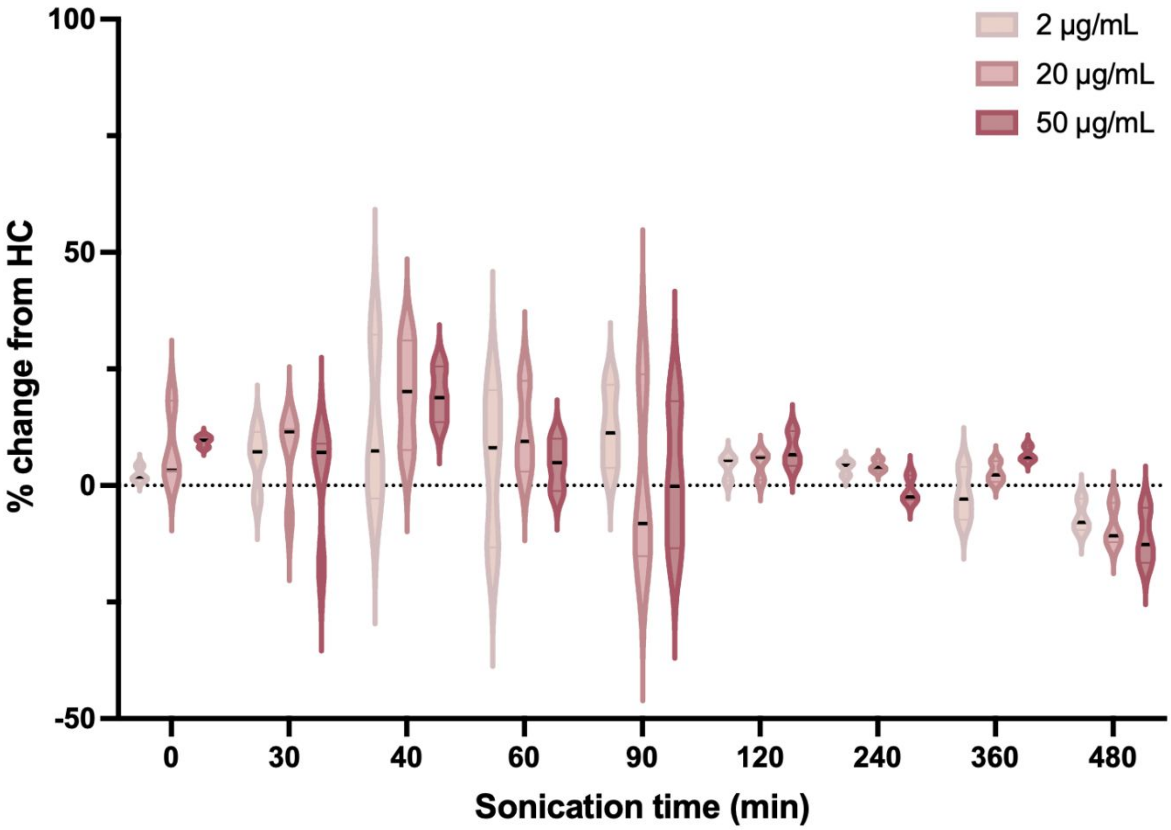

Figure S14. Change in metabolic activity of healthy BV-2 cells with the addition of 3 different doses of PNTs of different lengths generated by different sonication times compared to HC. Data are presented as violin plots that show the full range with lines at the median with interquartile range. (n = 3-4).

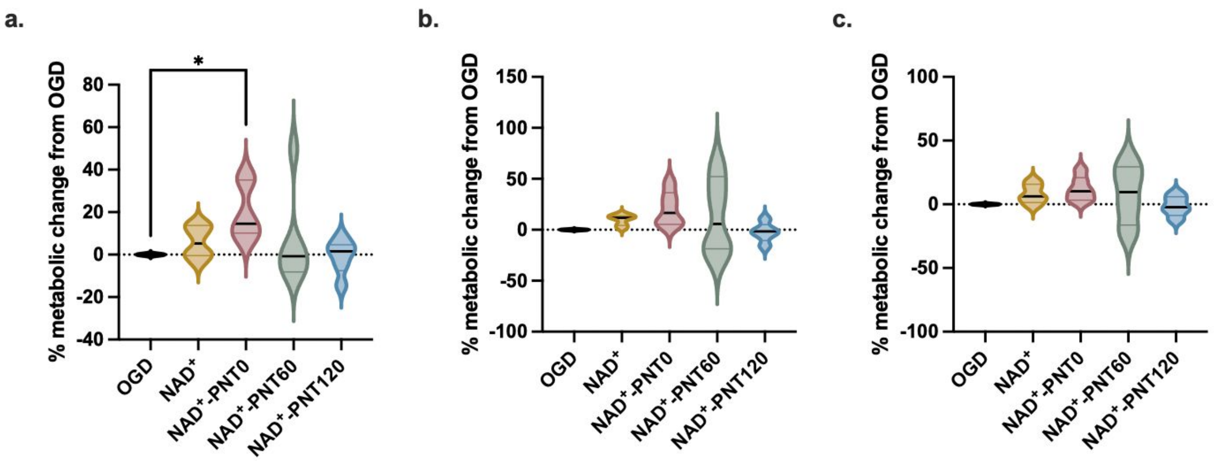

Figure S15. Sonication time impacts cell viability compared to non-treated OGD exposed BV-2 cell controls for 20µg/mL of NAD+ delivered via PNTs with a) 24 h, b) 48 h and c) 72 h. Statistical significance was calculated via a Kruskal-Wallis test. Data are presented as violin plots that show the full range with lines at the median with interquartile range. (n=4-6).

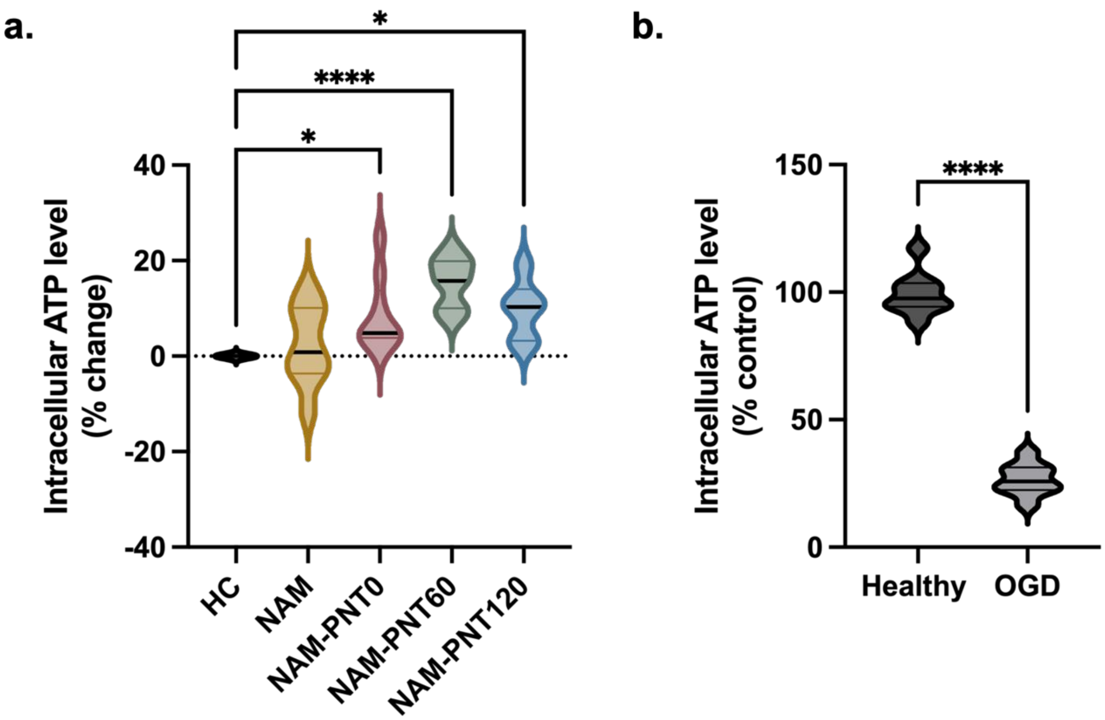

Figure S16. Intracellular ATP. a) Healthy BV-2 cells treated with NAM-PNTs showed an increase in intracellular ATP. b) Intracellular ATP is decreased in response to OGD-exposed in BV-2 cells. Statistical significance was calculated via a Kruskal-Wallis test. Data are presented as violin plots that show the full range with lines at the median with interquartile range. (n = 6-9).

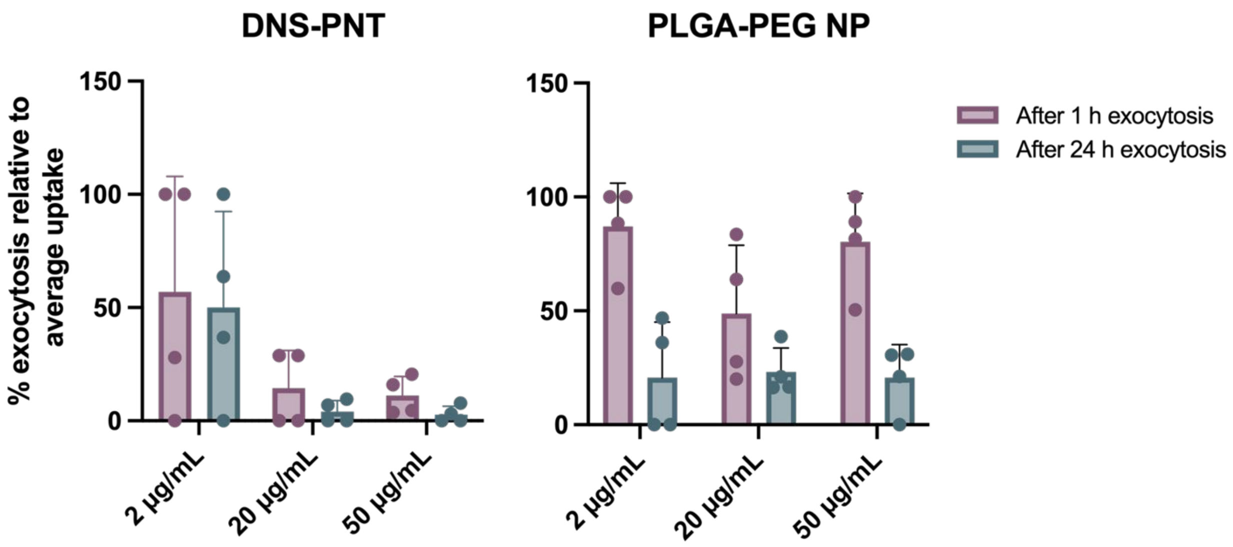

Figure S17. Exocytosis (1h or 24 h) of DNS-PNT and PLGA-PEG NPs (percentage) relative to the average uptake of cellular association (0–1 h) in the BV-2 cell model. Data are presented as mean ± s.d. (n = 6).

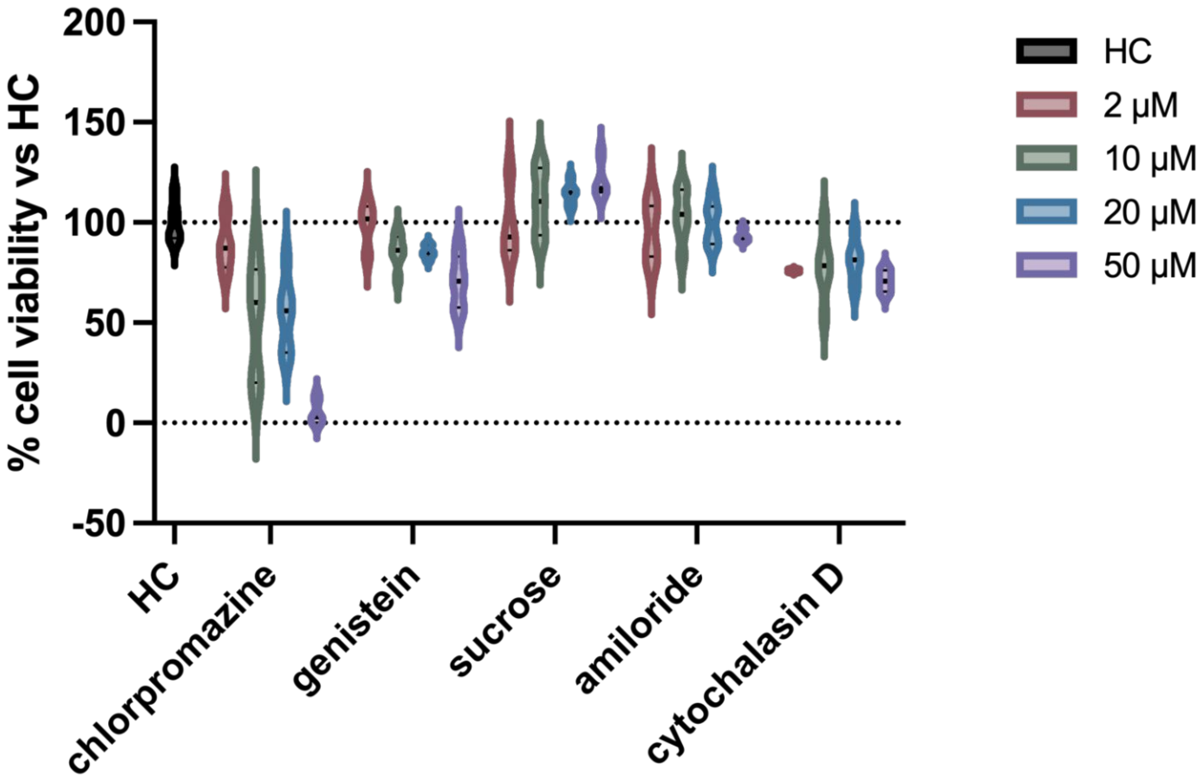

Figure S18. MTT data showing BV-2 cell viability after treatment with inhibitors for 24 hours at different concentrations. Data are presented as violin plots that show the full range with lines at the median with interquartile range. (n =4-6).

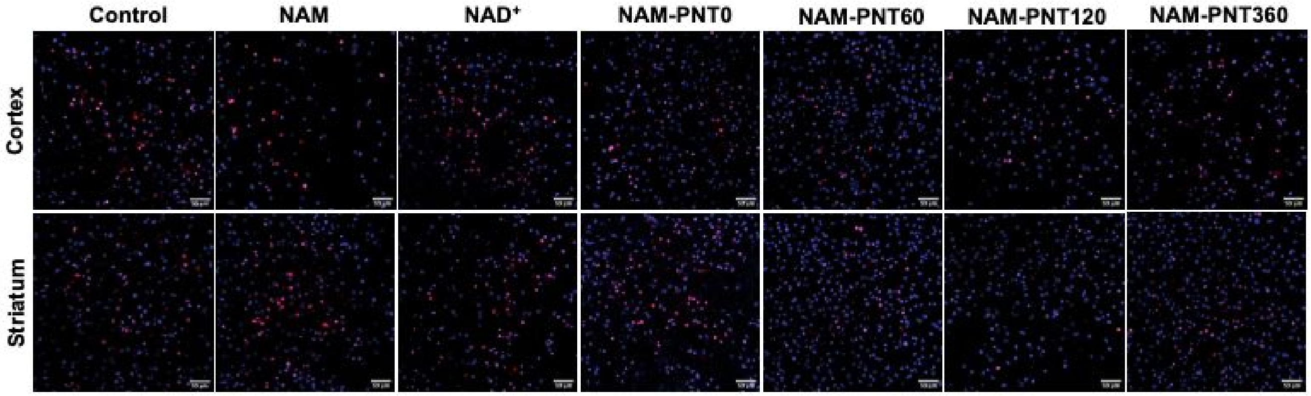

Figure S19. Representative images of propidium iodide signal (red) to visualize the amount of necrotic cell death after OGD or with 24 h of NAM, NAD, or NAM-PNT treatment applied immediately after OGD. Nuclei are stained with DAPI (blue). Scale bar, 50 μm.

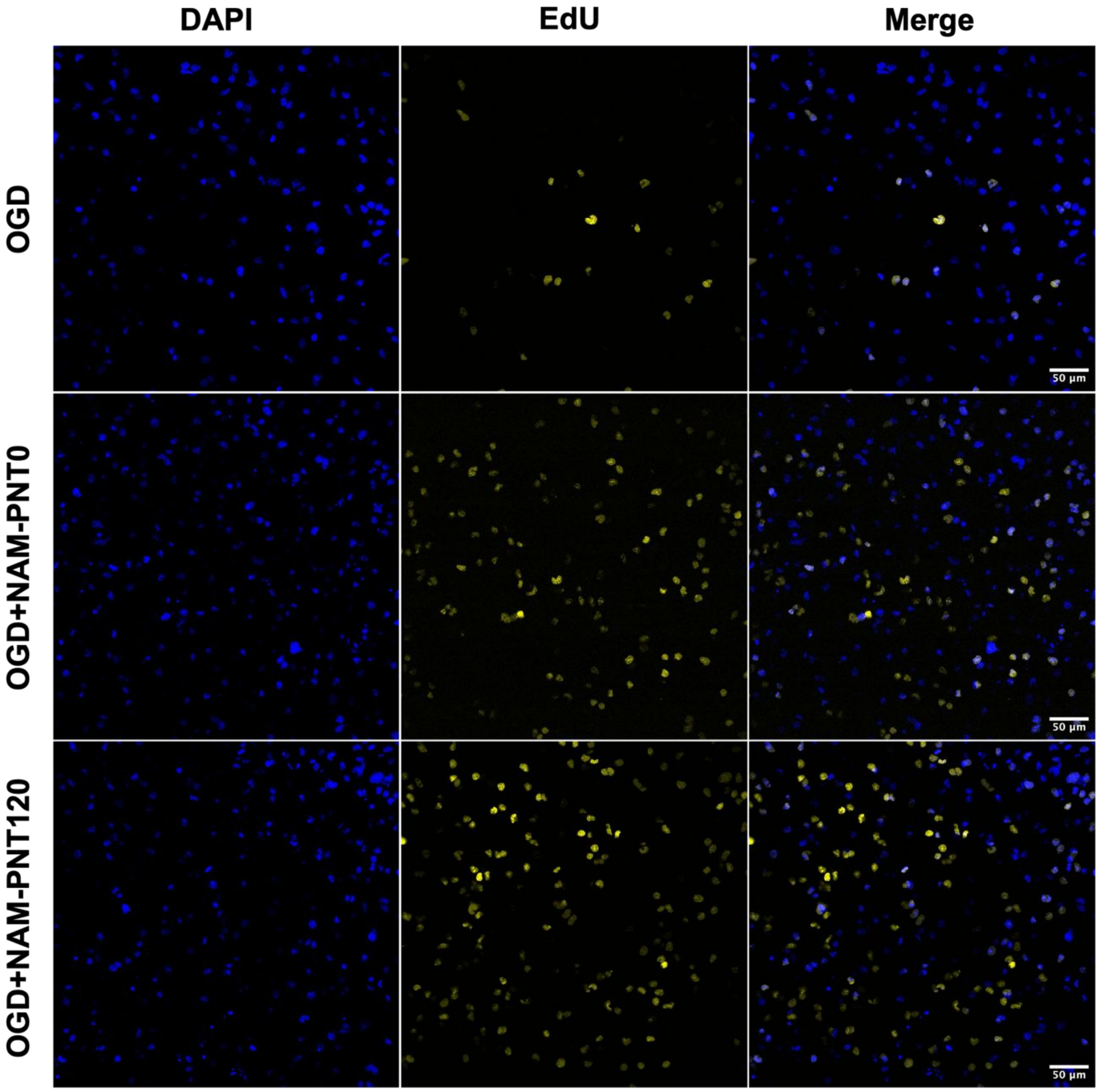

Figure S20. Representative images of EdU+ cells (yellow) 24 h after 30 min OGD at 4DIV from P10 slices. Nuclei are stained with DAPI (blue). Scale bar, 50 µm.

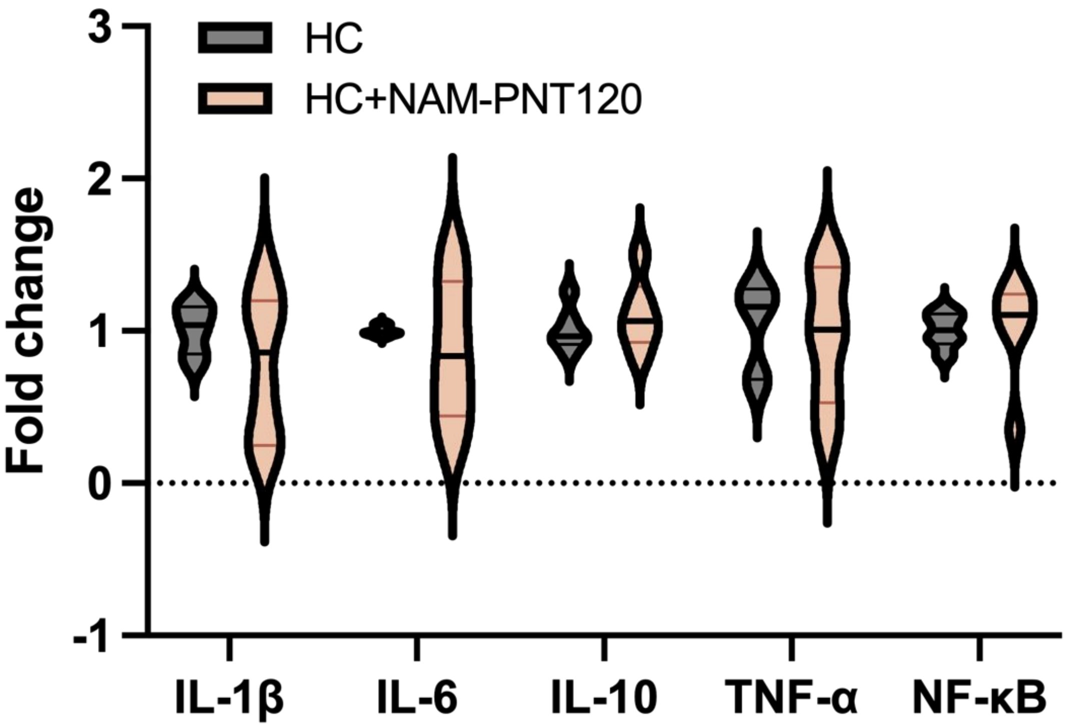

Figure S21. Fold-changes of mRNA markers for healthy slices with and without NAM-PNT treatment. Data are presented as violin plots that show the full range with lines at the median with interquartile range.

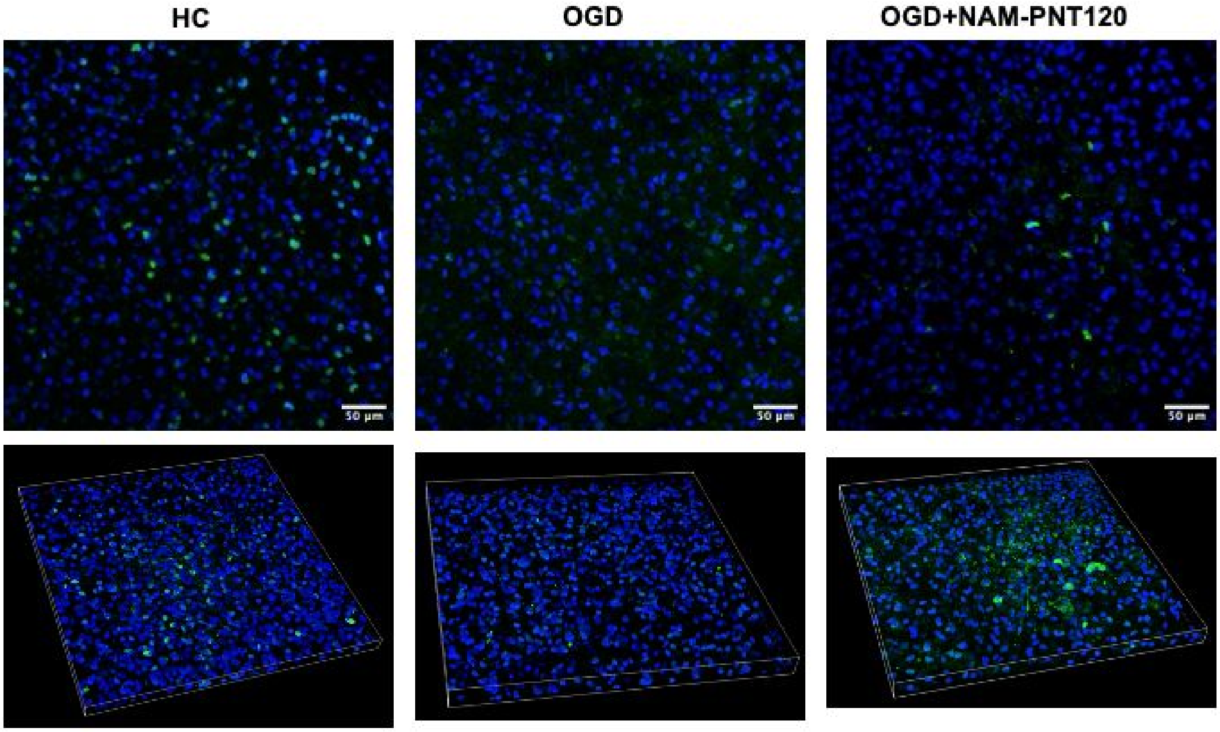

Figure S22. Representative images of PAR signal (green) shows the extent of DNA damage in healthy, OGD, and OGD with NAM-PNT120 OWH brain slices. Nuclei are stained with DAPI. Scale bar, 50 μm.

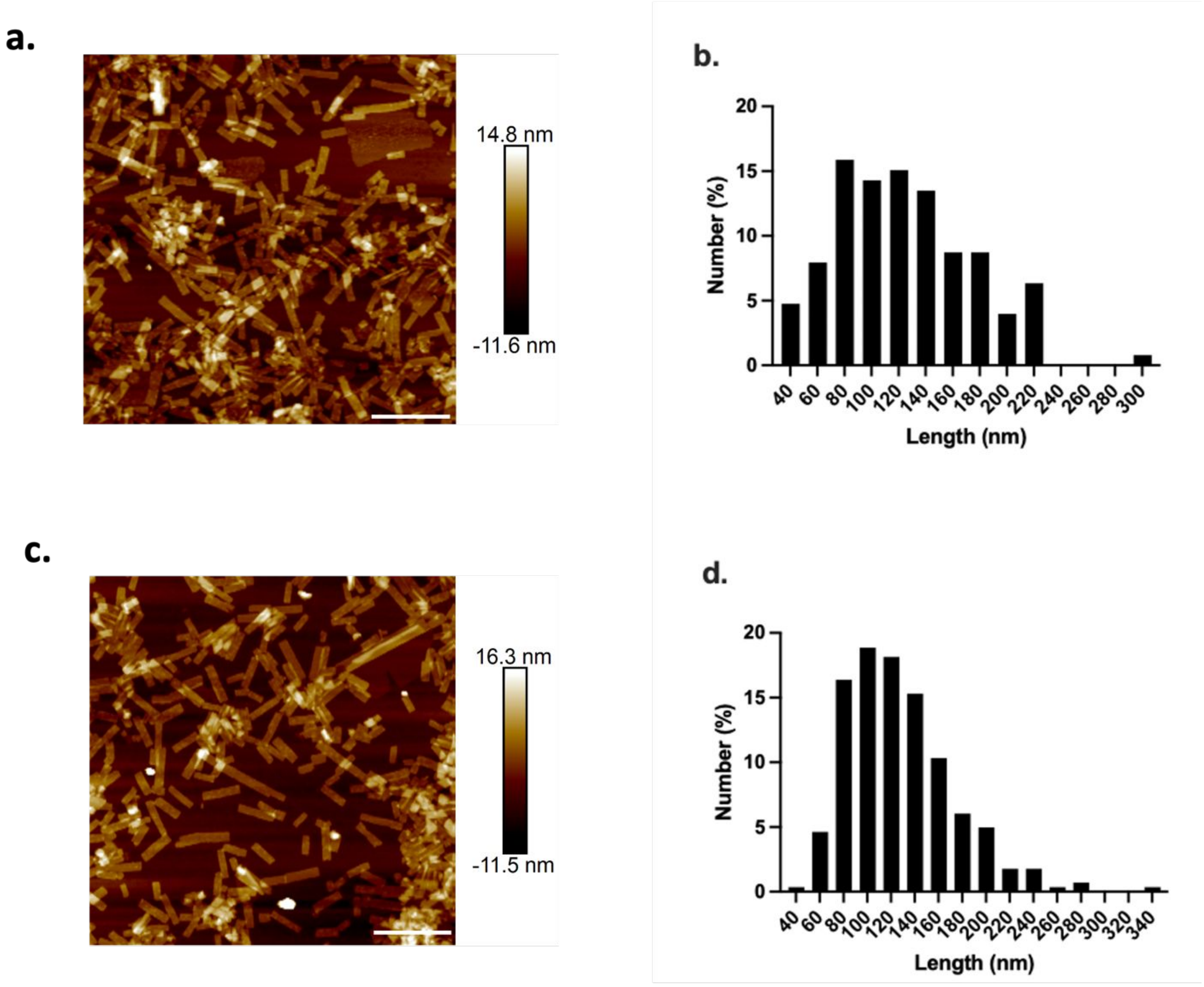

Figure S23. Representative AFM images and size distribution of a-b) PNT and c-d) NAM-PNT use for the in vivo study in HI injured rat pups. With 10 minutes of sonication-based emulsification, PNT has length of 124.24 ± 49.83 nm and NAM-PNT of 128.16 ± 48.35 nm. Scale bar, 200 nm.

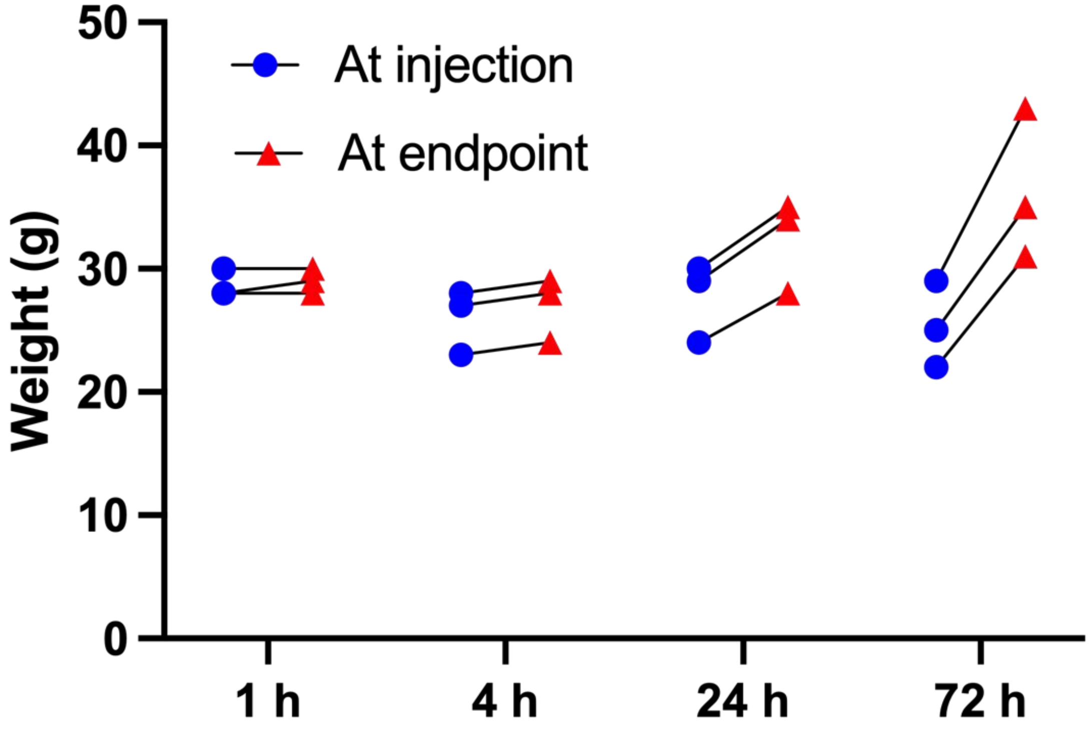

Figure S24. Weight change of healthy P10 rats before and after PNT injection up to 72 h. (n=3 per timepoint).

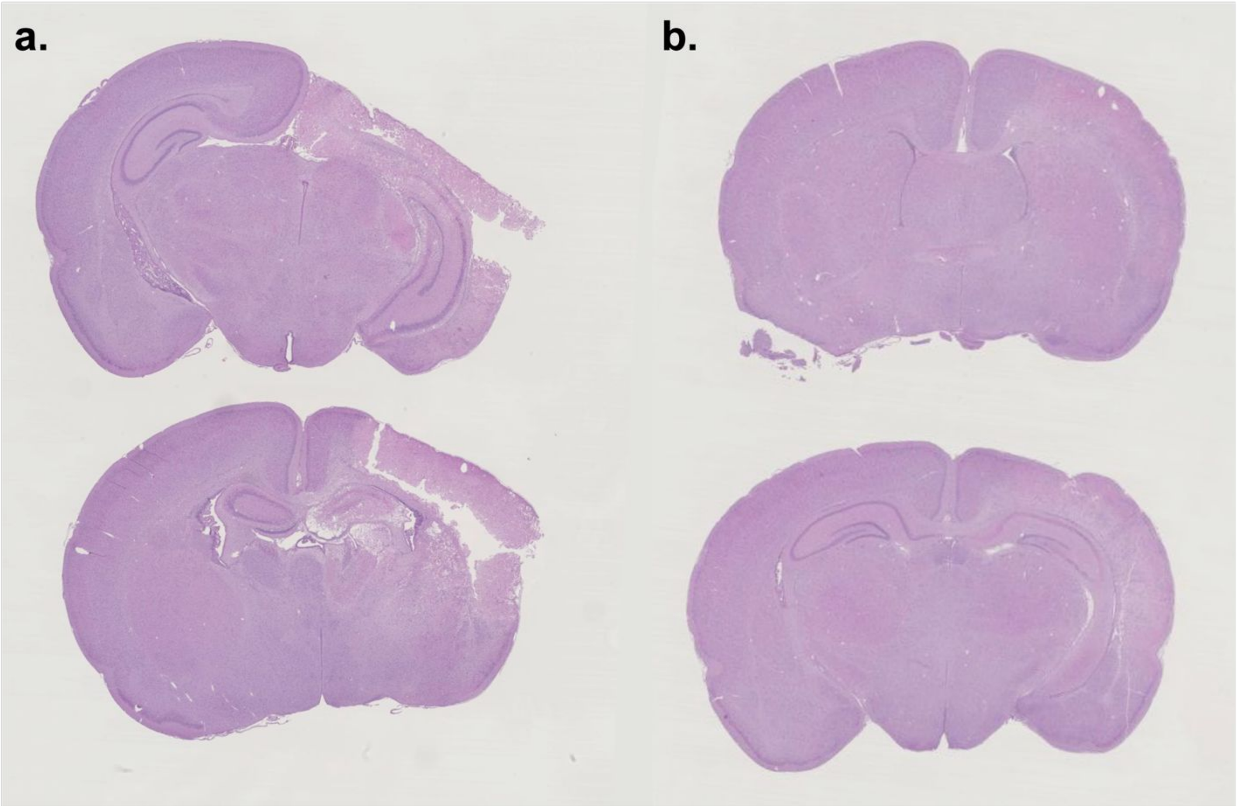

Figure S25. Representative images from a) Vehicle and b) NAM-PNT pups exposed to HI at P10. Increased percent area loss was observed in the vehicle group compared to the NAM-PNT group. Brains are from the media animal for each experimental group and are both female brains.

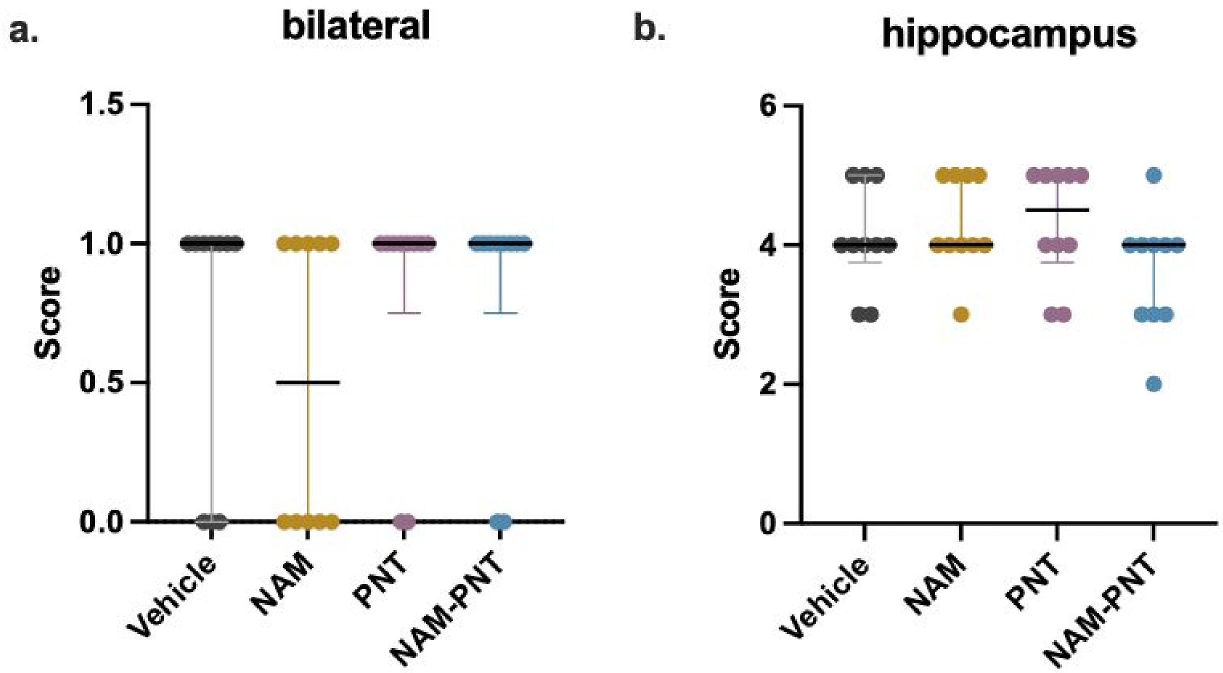

Figure S26. a) bilateral injury from pathology (score of 1) or unilateral injury (score of 0). b) hippocampus neuropathology data score of 0-5. Data are presented as scatter dot plots with lines at the median with interquartile range.

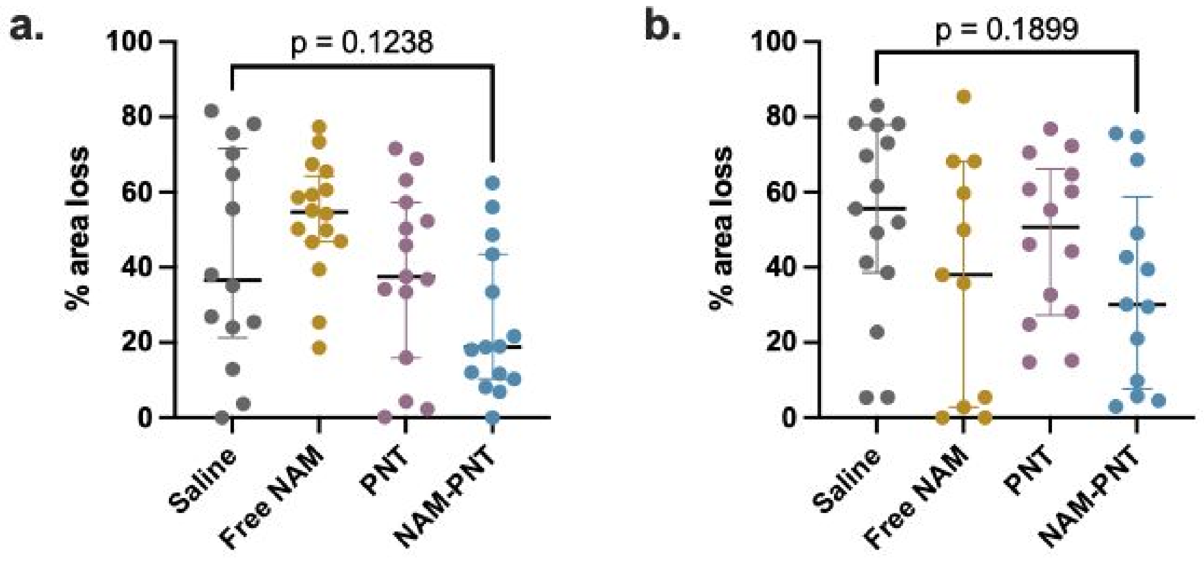

Figure S27. Total area of loss of a) male and b) female were calculated by assessing the percent area of tissue lost in the ipsilateral hemisphere, normalized to the contralateral hemisphere. Statistical significance was calculated via a Kruskal-Wallis test. Data are presented as scatter dot plots with lines at the median with interquartile range.

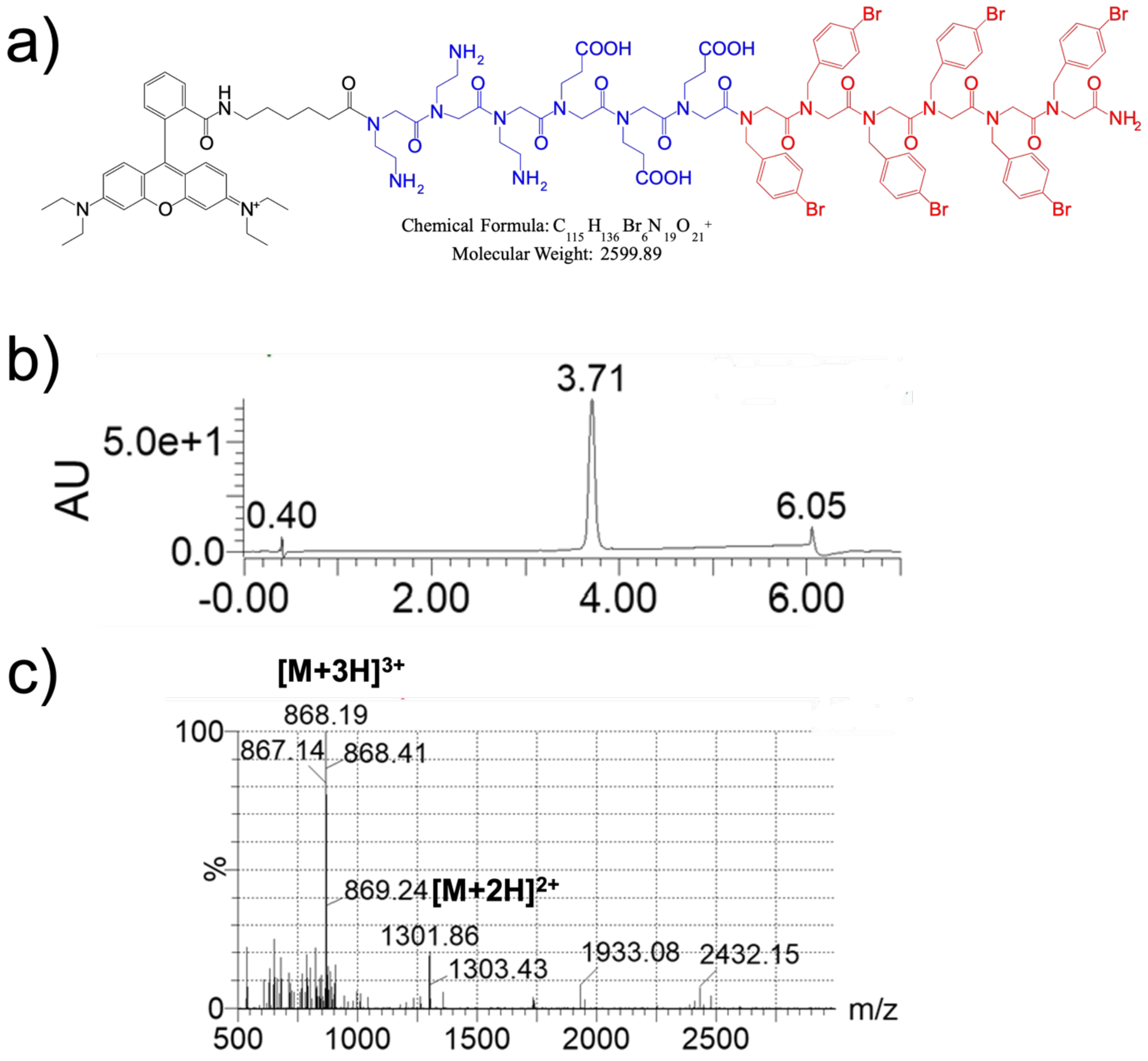

Figure S28. LC-MS analysis of Rhd-PNT. a) Chemical structure of PNT. b) LC profile of PNT run under gradient of acetonitrile from 5 -95 %. c) ESI mass analysis of PNT.

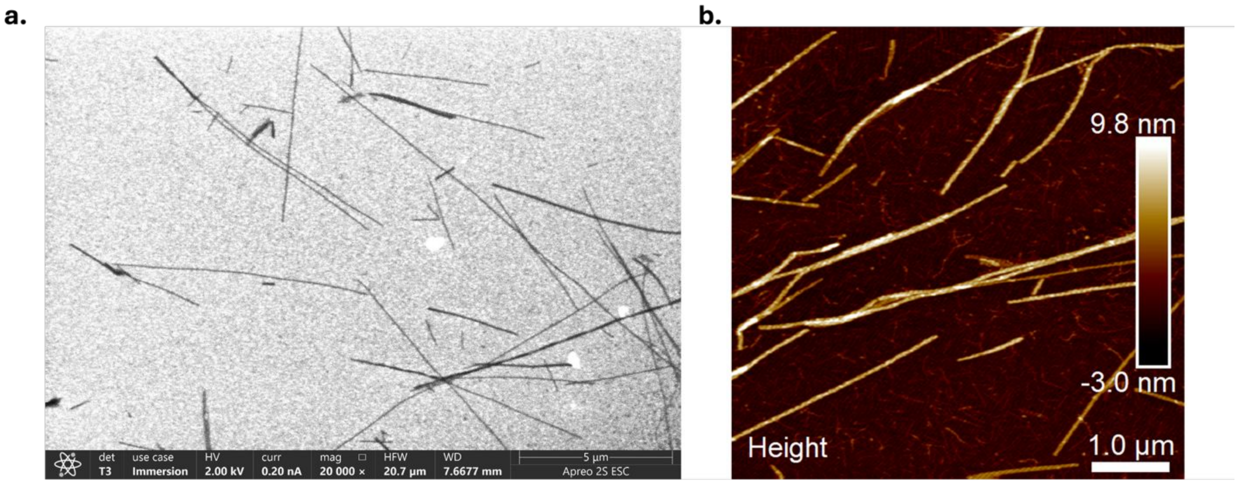

Figure S29. a) Representative AFM images of Rhd-PNT, respectively. b) Representative SEM images of Rhd-PNT.

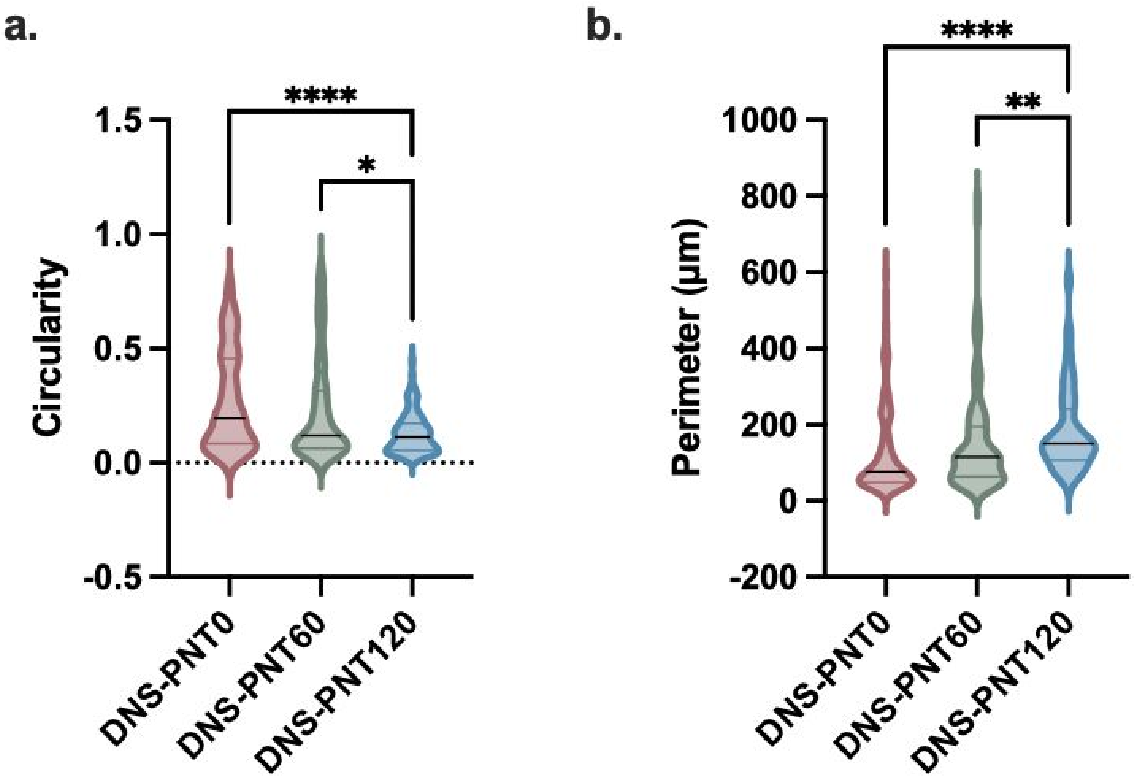

Figure S30. Microglia morphological analysis of a) circularity and b) perimeter in healthy OWH slices after exposure to DNS-PN0, DNS-PNT60 and DNS-PNT120 for 24 h. Statistical significance was calculated via a Kruskal-Wallis test. Data are presented as violin plots that show the full range with lines at the median with interquartile range.

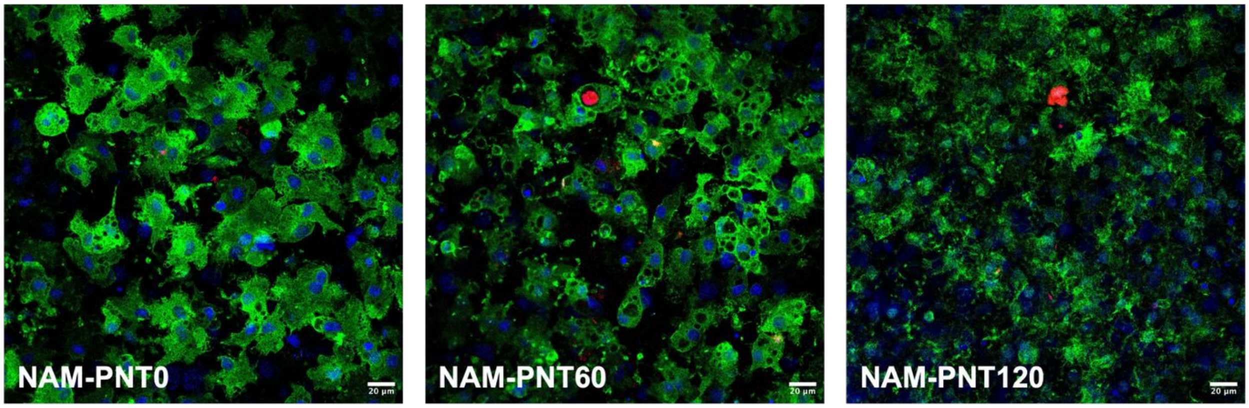

Figure S31. DS-PNT (red) localization in Iba1+ microglia (green) and ToPro-3 stained nuclei (blue) in OGD-exposed slices. Scale bar, 20 µm.

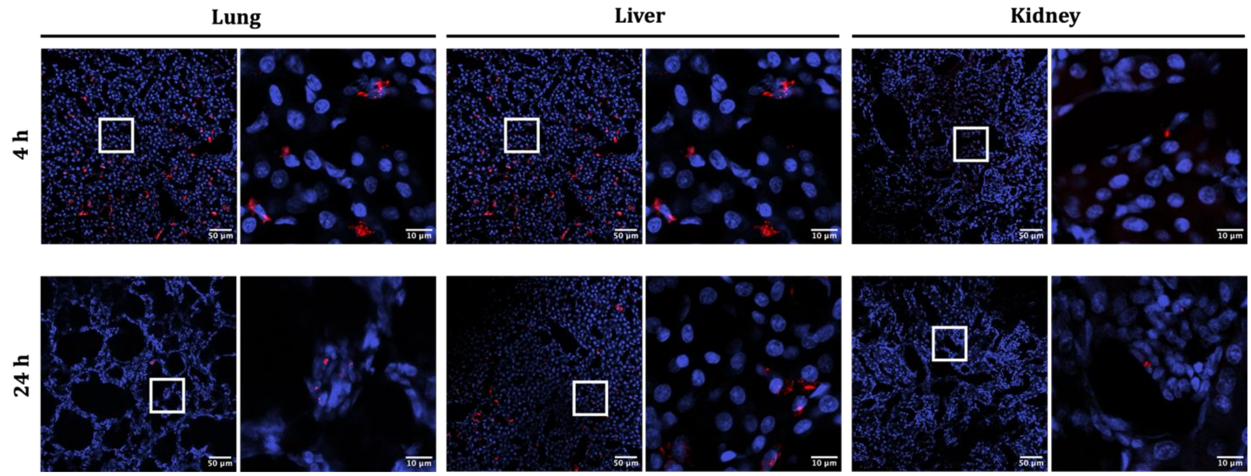

Figure S32. Representative images of Rhd-PNTs (red) localization in DAPI (blue) stained nucleus in lung, liver, and kidney in the HI-treated P10 rats after 4 h and 24 h of administration (40x and 240x).

Table S1. Average NAM-PNTs length and aspect ratio under different sonication times.

	0 min	30 min	60 min	120 min	240 min	360 min	480 min
Ave length (nm)	2343.61 ± 2249.96	561.29 ± 699.26	534.00 ± 479.58	360.78 ± 261.05	103.60 ± 47.45	102.13 ± 49.80	102.93 ± 51.41
Aspect ratio	63.00	15.09	14.35	9.70	2.79	2.75	2.77

Table S2. List of primers use for RNA analysis in ex vivo samples.

Target	Forward Primer	Reverse Primer
GAPDH	ACTCCCATTCTTCCACCTTTG	CCCTGTTGCTGTAGCCATATT
IL-1β	CTATGGCAACTGTCCCTGAA	GGCTTGGAAGCAATCCTTAATC
IL-6	GAAGTTAGAGTCACAGAAGGAGTG	GTTTGCCGAGTAGACCTCATAG
IL-10	AGTGGAGCAGGTGAAGAATG	GAGTGTCACGTAGGCTTCTATG
TNF-α	GCCGATTTGCCACTTCATAC	AAGTAGACCTGCCCGGACTC
NB-κB	GGTTACGGGAGATGTGAAGATG	GTGGATGATGGCTAAGTGTAGG
